# First record of the subfamily Epitraninae from Saudi Arabia (Hymenoptera, Chalcidoidea, Chalcididae), with the description of three new species

**DOI:** 10.3897/zookeys.979.52059

**Published:** 2020-10-27

**Authors:** Neveen S. Gadallah, Ahmed M. Soliman, Hathal M. Al Dhafer

**Affiliations:** 1 Entomology Department, Faculty of Science, Cairo University, Giza, Egypt Cairo University Giza Egypt; 2 Plant Protection Department, College of Food and Agriculture Sciences, King Saud University, P.O. BOX 2460, Riyadh 11451, Saudi Arabia King Saud University Riyadh Saudi Arabia; 3 Zoology Department, Faculty of Science (Boys), Al-Azhar University, P.O. Box 11884, Nasr City, Cairo, Egypt Al-Azhar University Cairo Egypt

**Keywords:** Afrotropical, Arabian Peninsula, *
Epitranus
*, new records, new species

## Abstract

The monotypic subfamily Epitraninae Burks, 1936 (Hymenoptera: Chalcidoidea, Chalcididae) is reported for the first time in Saudi Arabia. Seven *Epitranus* species are recorded in the Southwestern and Central regions of the Kingdom of Saudi Arabia, of which three species are new: *E. delvarei* Soliman & Gadallah, **sp. nov.** (female & male), *E. similis* Gadallah & Soliman, **sp. nov.** (male), and *E. subinops* Soliman & Gadallah, **sp. nov.** (female), are described and illustrated. Four new records, *E. clavatus* (Fabricius), *E. hamoni* complex, *E. inops* Steffan, and *E. torymoides* (Risbec), are also reported. An illustrated key to species is provided.

## Introduction

Epitraninae Burks was first treated as tribe Chalcitellini by Ashmead (1904) with the type genus *Chalcitella* Westwood. This tribe was renamed Epitranini by Burks (1936) when the genus *Chalcitella* was treated as a junior synonym of *Epitranus* Walker. Habu (1960) raised Epitranini to the subfamily rank, Epitraninae, based on the fact that this group differs completely in its morphological characters from any other tribal taxa. The controversial taxonomic history of Epitraninae is well discussed by Bouček (1982). From the phylogenetic point of view, Epitraninae was treated as a tribe in the subfamily Dirhininae by Heraty et al. (2013) based on both morphological and molecular data. On the other hand, a recent phylogenetic study was carried out by Cruaud et al. (2020), treated it as a separate subfamily among the family Chalcididae, based on recent morphological and molecular data with novel computational approaches.

Members of Epitraninae are easily recognized by the following combination of characters: absence of cephalic horns; antennae inserted at lowermost part of face, very near to oral fossa on a protrusion or, most often, on a protruding lobe of frons “frontal lobe” (wrongly named clypeus by some authors), masking clypeus; frontal lobe with its free margin either rounded or denticulate, and may be divided by inter-antennal lamella; frons more or less flat; gena with strong posterior carina that extends into a flange; mesoscutellum simple, strongly convex and rounded at posterior margin; propodeum horizontal, its sculpturing clear and well-marked, areola often present medially; tegula flattened sometimes extends into a flange posteriorly to overlap base of hind wing; marginal vein of fore wing extremely long relative to the short stigmal vein and the reduced or absent postmarginal vein; metafemur with a comb of contiguous small teeth or spaced teeth following a large more or less triangular basal tooth; metatibia ending in a curved tibial spine, with a distinct tarsal scrobe, varying in length, that extends to reach a proximal sub-basal prominence; metasoma with a long narrow, striated petiole, several times as long as wide, or in some cases may be longer than half length of the gaster; gastral body rather small, compressed from side-to-side, and bulging ventrally, first gastral tergite occupying almost the total part of metasoma, thus mostly concealing the remaining tergites (Steffan 1957; Bouček 1982, 1988; Husain and Agarwal 1982; Narendran and van Achterberg 2016; Delvare 2017).

Sexual dimorphism is only slight (Bouček 1982). In the female, the metasoma is somewhat acuminate distally, with a pair of short dark ovipositor sheaths, the hypopygium ends at a short distance before the ovipositor sheaths (in male, the metasoma is shorter and blunt distally); in female the antenna shorter, more or less clavate, with apical flagellomeres shorter (in male, the antenna longer and filiform, with longer flagellomeres) (Bouček 1982).

All Epitraninae are now classified in a unique genus, *Epitranus* Walker (Bouček 1982, 1988; Narendran and van Achterberg 2016; Delvare 2017; Noyes 2019), which in turn comprises currently a total of 68 described species (Noyes 2019). The genus had been pulverized into several genera (see Bouček 1982, 1988), latter considered as synonyms by Burks (1936) and then Bouček (1982).

Little is known about the biology of Epitraninae. Hosts are known from only seven Oriental (Bouček 1982, 1988; Narendran 1989; Narendran and van Achterberg 2016; Noyes 2019), and a single Afrotropical species (Sauphanor et al. 1987). All of them parasitizing small lepidopteran moths of the families Crambidae, Pyralidae and Tineidae (Bouček 1982, 1988; Sauphanor et al. 1987; Narendran 1989; Narendran and van Achterberg 2016; Noyes 2019). This is in addition of two records, *E. chilkaensis* (Mani) reared from a nest of *Camponotus compressus* (Formicidae) (Narendran 1989) and *E. emissicius* Steffan that was found as living in subterranean nests of *Mastotermes* sp. (Mastotermitidae) (Rasplus 1993). Other few species are reported as having economic importance attacking lepidopteran pests infesting stored products (Sauphanor et al. 1987). Adults can be seen on foliage of trees and shrubs, and collected from fonds of woody plants, but usually not on grass (Bouček 1982, 1988). The relatively large ocelli in some species suggests their activity at dusk or even at night (Bouček 1982).

More than half number of *Epitranus* species are Oriental in distribution (54.65%) (Bouček 1982; Narendran and van Achterberg 2016), followed by the Afrotropical region (38%) (Schmitz 1946 under *Anacryptus*; Steffan 1957; Noyes 2019) and very little are Australasian (7.35%) (Girault 1913, 1914, 1915). Burks (1936) suggested their presence even in the Nearctic region, based on the proximity of Florida to St. Vincent and Cuba, where *Epitranus* species were found.

Concerning the fauna of the Arabian Peninsula, the only work dealing with this group, was that by Delvare (2017) in his revision of the whole family (Chalcididae) in the United Arab Emirates. He reported two *Epitranus* species, *E. hamoni* (Risbec) and *E. torymoides* (Risbec).

The present study is the first attempt to study the Epitraninae of the fauna of the Saudi Arabia. Four new records, *E. clavatus* (Fabricius), *E. hamoni* complex, *E. inops* Steffan, and *E. torymoides* (Risbec), as well as three new species are described and illustrated, *E. delvarei* sp. nov., *E. similis* sp. nov. and *E. subinops* sp. nov. A key to separate the species, as well as faunistic list are also provided.

## Materials and methods

The present study is based on specimens collected from some mountains and wadis in Al-Baha, Asir and Jazan (southwestern regions of Saudi Arabia) and Riyadh (central region of Saudi Arabia) provinces (Fig. 31). Sampling was done by means of a sweeping net, vacuum machine (McCulloch GBV325 vacuum), and Malaise trap. Identifications of some species were done with the help of Gerard Delvare (Cirad, Montferrier-sur-Lez, France) during the visit of NG to Cirad, Montpellier. In addition, the authors used the keys and original descriptions of Schmitz (1946), Steffan (1957), and Bouček (1982). Morphological terms follow Bouček (1988). The terminology of body sculpture follows Harris (1979). Photographic images were taken using a Canon EOS 70D camera attached to a LEICA MZ-125 stereomicroscope. Individual source images were then stacked using HeliconFocus v6.22 (HeliconSoft Ltd) extended depth of field software. Measurements were made with the help of an ocular micrometer. Further image processing was done using the software Adobe Photoshop CS5.1 (ver. 12.1x32) and Adobe Photoshop Lightroom 5.2 Final [ChingLiu]. The type specimens of the new species are deposited in King Saud University Museum of Arthropods (**KSMA**), Plant Protection Department, College of Food and Agriculture Sciences, King Saud University, Riyadh, Saudi Arabia. A representative of *E. torymoides* is kept in Efflatoun’s Collection (EFC), Entomology Department, Faculty of Science, Cairo University, Giza, Egypt.

### Abbreviations

**AOL** = Distance between median and lateral ocelli; **F1, F2, F3, F7** = first, second, third & seventh funicular segments; **Gt** = gastral tergite; **MPS** = multiparous plate sensilla; **MV** = marginal vein of fore wing; **OD** = lateral ocellus diameter; **OOL** = distance between lateral ocellus and inner eye margin; **POL** = distance between lateral ocelli; **SMV** = submarginal vein; **STV** = stigmal vein.

## Systematic accounts

### *Epitranus* Walker, 1834

For a complete list of synonyms, see Bouček (1982, 1988).

#### Key to species of the genus *Epitranus* in Saudi Arabia (mostly based on females)

**Table:** 

1	*Both sexes*. Fore wing almost lacking venation, only base of SMV visible (Fig. 29C); flagellum between pedicel and clava with 7 flagellomeres, the two basal ones without MPS (Fig. 29B); frontal expansion not much developed, its ventral edge rounded and lacking indentations (Fig. 29A)	***Epitranus torymoides* (Risbec)**
–	*Both sexes*. Fore wing with complete venation (e.g., see Fig. 3C); flagellum with 8 flagellomeres between pedicel and clava, only the transverse basal one (anellus) lacking MPS (e.g., Fig. 2D); frontal expansion absent (e.g., see Fig. 2B) or well developed and distinctly protruding (e.g., see Fig. 7B)	**2**
2	*Both sexes*. Metafemur ventrally with at least 11 teeth following the large basal tooth, all teeth small, similar and contiguous (e.g., see Fig. 4A, B)	**3**
–	*Both sexes*. Metafemur ventrally with at most 9 teeth following the large basal one, the sub-basal teeth relatively larger than in alternate and more widely spaced (e.g., see Fig. 8D, E)	**5**
3	Metatibia with oblique carina inside metatibial process (Fig. 13B); tarsal scrobe almost reaching sub-basal prominence (Fig. 13B); frons with supra antennal surface completely delimited by a step-like margin; mesoscutum with short setae and very small and sparse punctures on anterior third of middle lobe (Fig. 11E); bottom of punctures on mesonotum and metepimeron smooth (Figs 11E, 12B)	***Epitranus subinops* sp. nov .**
–	Metatibial process without such carina (Figs 4B, 27C); tarsal scrobe shorter (Fig. 27C) or bottom of punctures granulate (densely reticulate) on mesonotum and metepimeron (Figs 2E, 3B); supra antennal surface at most delimited laterally, sometimes undifferentiated; mesoscutum with setae longer and punctures denser and coarser on the whole middle lobe (Figs 2E, 25B)	**4**
4.	Bottom of punctures granulate (densely reticulate) on mesoscutellum and metepimeron (Figs 2E, 3B); interspaces between punctures coriaceous (engraved network) on mesoscutum (Figs 2E, 3B); metatibia with tarsal scrobe almost reaching sub-basal prominence (Fig. 4B); mesoscutellum convex, its dorsal outline curved in lateral view (Figs 2E, 3B); no differentiated supra antennal surface	***Epitranus delvarei* sp. nov.**
–	Bottom of punctures and interspaces smooth on mesonotum and metepimeron (Fig. 25A, B); tarsal scrobe of metatibia far from reaching sub-basal prominence (Fig. 27C); mesoscutellum flattened, its dorsal outline straight in lateral view (Fig. 25A, B); supra antennal surface delimited laterally by faint step-like margin	***Epitranus inops* Steffan**
5	*Both sexes*. Frontal expansion reduced to a transverse carina, hence clypeus visible in frontal view (Figs 18B, 22A); frons entirely densely and faintly reticulate and bearing very short, hardly discernible setae (Figs 18A, 22A); whole mesosoma, including shallow punctures and interspaces on mesonotum, bottom of areolae on propodeum, granulate (densely reticulate), thus appearing dull (Figs 18D, 20B, 21B, 24B), and metatibia with extremely weak sub-basal prominence (Fig. 19B); head and mesosoma partly testaceous (Figs 18A, D, 20B, 21A, B). *Female*. Interantennal projection expanded only as a small lamina. *Male*. Scape with deep and setose sub-basal excavation but lacking any dorsal row or patch of setae (Figs 22B, 24A)	***Epitranus hamoni* complex**
–	*Both sexes*. Frontal expansion clearly expanded, overlapping clypeus (Figs 7B, 15B); upper frons and adorbital area alutaceous and with setiferous punctures (Figs 7A, 15A); interspaces or bottom of punctures on mesonotum smooth (Figs 7E, 14B), and metatibia with evident sub-basal prominence (Figs 8E, 16B); head and mesosoma with different pattern of color, partly reddish (Figs 7A, E, 14A, B). *Female*. Interantennal projection either expanded as a raised lamina (Fig. 15A) or completely absent (Fig. 7B). *Male*. Scape without such excavation but frequently with a row or brush setae dorsally (Fig. 6A)	**6**
6	*Male*. Frontal expansion quite protruding with subantennal distance 3.7–4.5× as long as interantennal distance (Fig. 7B); expansion sub-trapezoidal in shape as its sides are straight and regularly converging ventrally (Fig. 7B); expansion otherwise bearing thick, lanceolate and whitish setae on either side of median carina, with deep submedian indentations on ventral edge (Fig. 7B); interantennal lamina absent (Fig. 7B); long, lanceolate and golden setae present on occiput (here sparsely) (Fig. 7C) and on pronotal collar (here as a double patch) (Fig. 7C); mesosoma with patches of long, lanceolate and silvery setae on pronotum above lateral panel, scapula, axilla, metepimeron, and on pre-spiracular areola of propodeum (Figs 7E, 8B) and with dense setation masking integument beneath mesosoma and metacoxa (Fig. 6C); pronotal lateral carina extended dorsally on collar (Fig. 7E); propodeum with a Y-like raised carina mesally (Fig. 8A); metatibia with tarsal scrobe deep and smooth throughout, clearly reaching sub-basal prominence (Fig. 8E), metatibial process without oblique carina (Fig. 8E)	***Epitranus similis* sp. nov.**
–	*Both sexes*. Frontal lobe less protruding than in alternate with subantennal distance ca. 1.7× as long as interantennal distance, without median longitudinal carina and lacking such setation, submedian indentations shallow and sides of expansion very slightly convex (Fig. 15B); interantennal lamina present (Fig. 15B); mesosoma without the setation as described above, the setae everywhere short and hair-like (Fig. 14A, B); pronotal carina restricted to sides (Fig. 14B); propodeum with median areola complete, somewhat tapering anteriorly (Fig. 15D); tarsal scrobe of metatibia not quite reaching sub-basal prominence and metatibial process with an oblique carina (Fig. 16C)	***Epitranus clavatus* (Fabricius)**

#### Description of the new species

##### Epitranus delvarei

Taxon classificationAnimaliaHymenopteraChalcididae

Soliman & Gadallah sp. nov.

1134283F-A6D8-57CF-83A3-F4AAE38B9427

http://zoobank.org/FC3FE0A6-CA05-4FBC-A6B1-85EF87E13E0C

**Figure 1.**
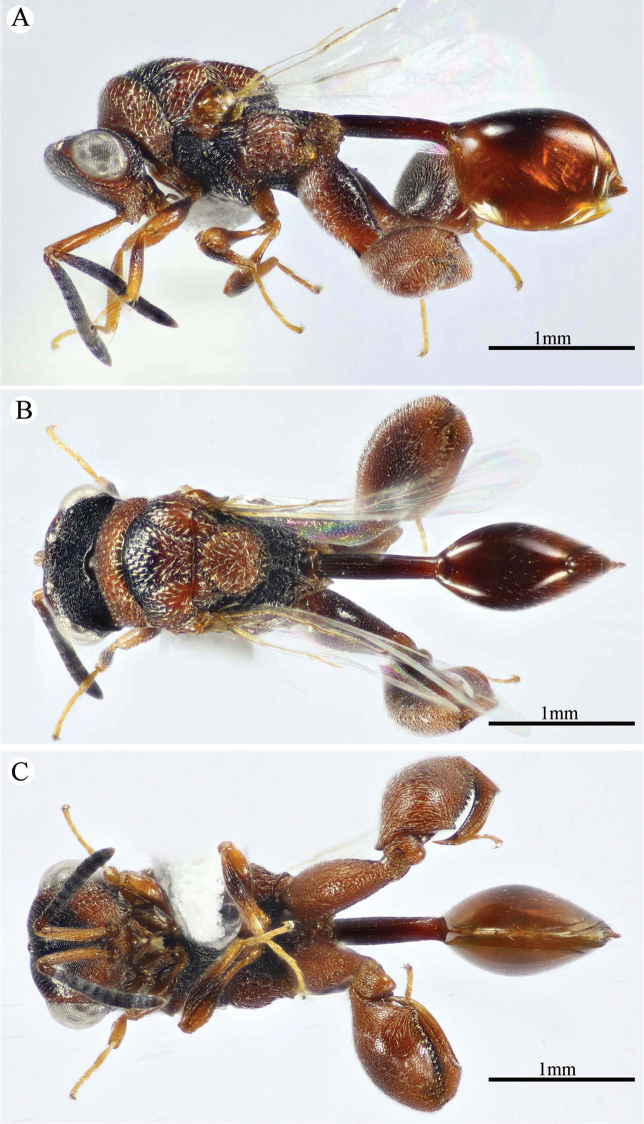
*Epitranus delvarei* Soliman & Gadallah, sp. nov. (holotype female). **A, B, C** habitus (lateral, dorsal and ventral views respectively).

**Figure 2.**
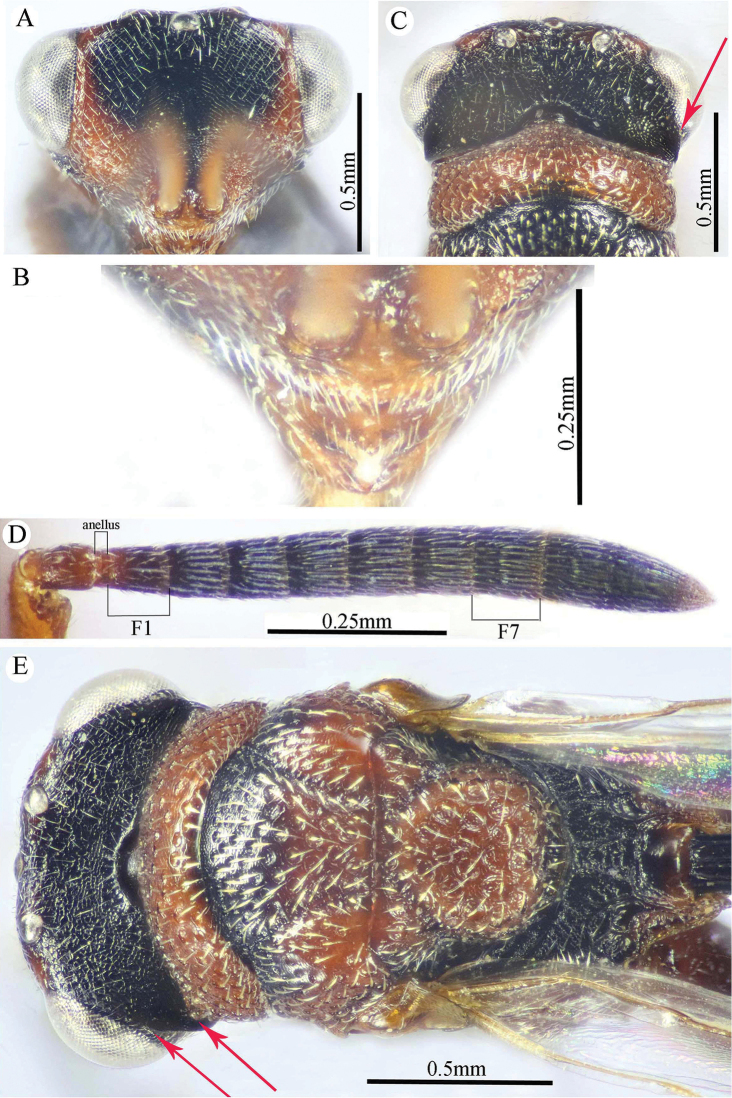
*Epitranus delvarei* Soliman & Gadallah, sp. nov. (holotype female) **A** head (frontal view) **B** lower part of face (frontal view) **C** head, pronotum & part of mesoscutum (dorsal view) **D** antennal pedicel and flagellum **E** head and mesosoma (dorsal view).

**Figure 3.**
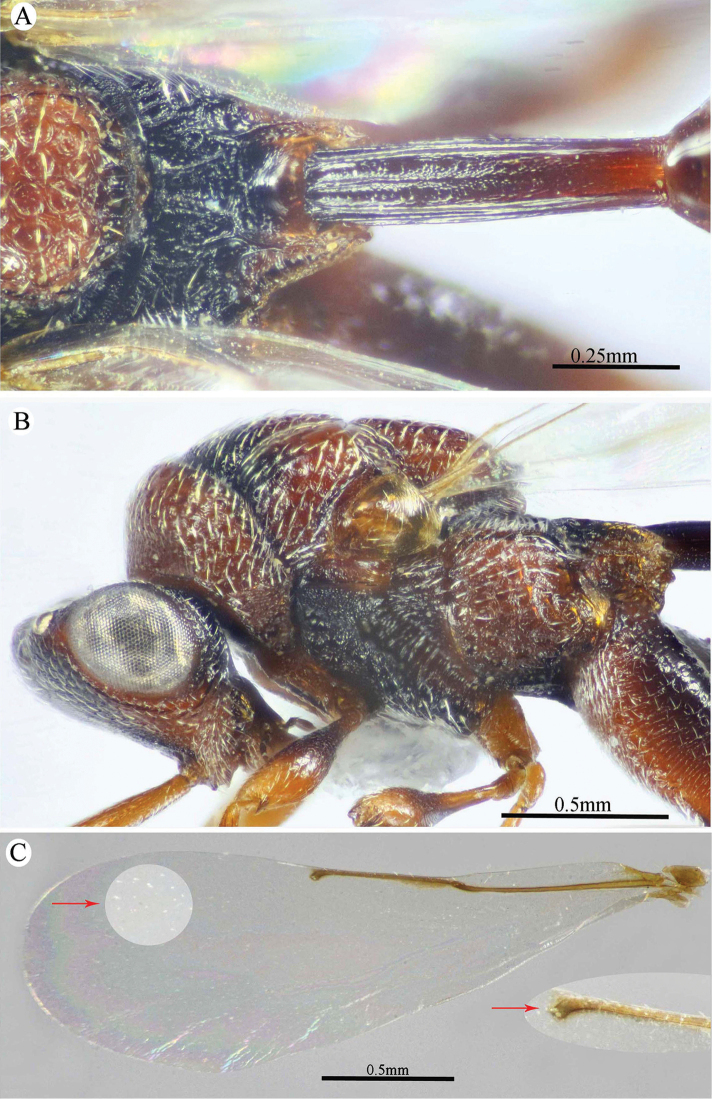
*Epitranus delvarei* Soliman & Gadallah, sp. nov. (holotype female) **A** part of mesoscutellum, metanotum, propodeum & gastral petiole (dorsal view) **B** head & mesosoma (lateral view) **C** fore wing (parts of wing membrane and MV and STV magnified).

**Figure 4.**
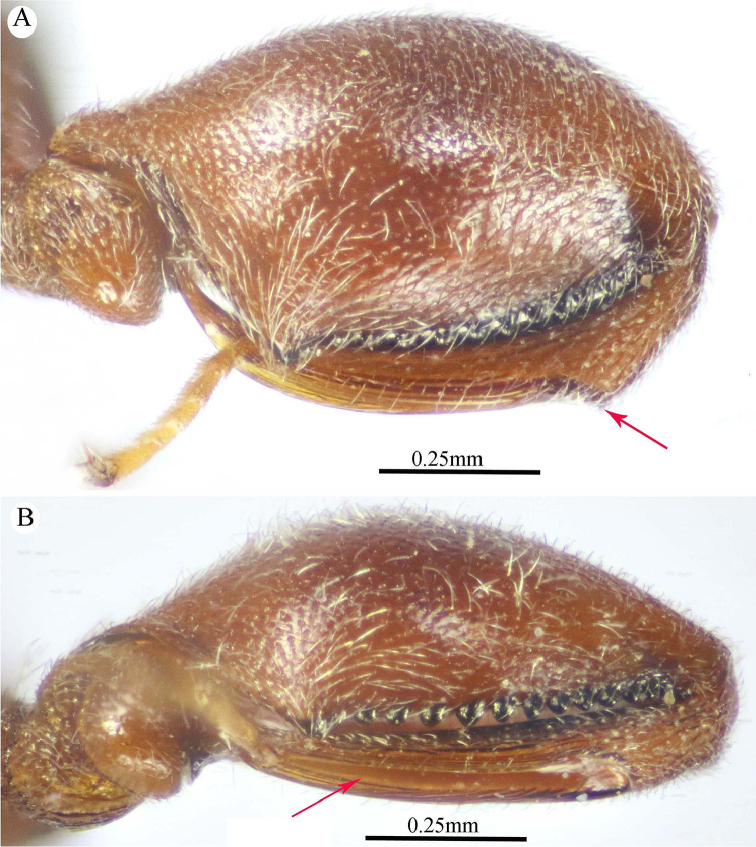
*Epitranus delvarei* Soliman & Gadallah, sp. nov. (holotype female) **A, B** hind leg, excluding coxa (outer and ventral views respectively).

**Figure 5.**
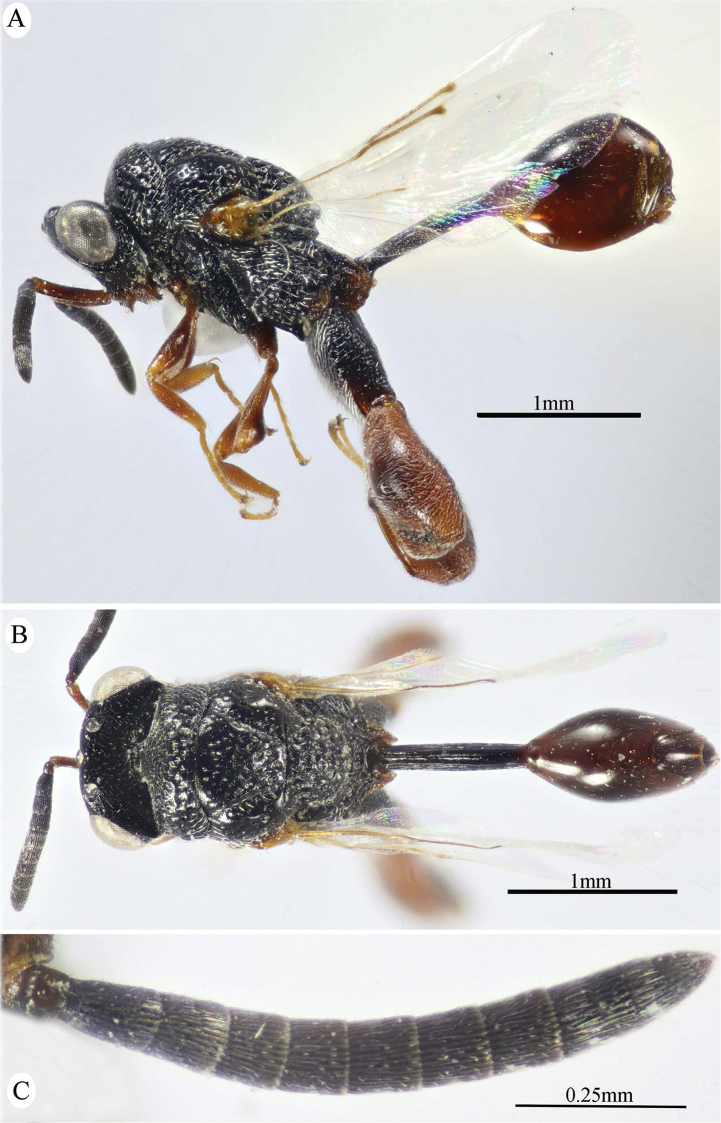
*Epitranus delvarei* Soliman & Gadallah, sp. nov. (paratype male) **A, B** habitus (lateral and dorsal views respectively) **C** antennal pedicel and flagellum.

Figures 1 , 2 , 3 , 4 , 5

###### Type material.

***Holotype*** ♀: **Kingdom of Saudi Arabia**, Asir, Abha, Garf Raydah Natural Reserve [18°11'41"N, 42°23'45"E, Alt. 1865 m], sweeping net, 12.IV.2019, leg. Ahmed M. Soliman [KSMA]. ***Paratypes***: 1♀, same data as for holotype [KSMA]; 2♂, **Kingdom of Saudi Arabia**, Al-Baha, Al Mikhwa, Shada Al-Ala Natural Reserve [19°50'34.95"N, 41°18'40.04"E, Alt. 1679 m], sweeping net, 7.IV.2019, leg. Ahmed M. Soliman [KSMA].

###### Diagnosis.

Frontal lobe short, entire at free margin (Fig. 2B); frons finely reticulate (Fig. 2A); supra-antennal surface absent (Fig. 2A); OOL slightly longer than AOD, ca. 1.75× OD (Fig. 2C); scape ends just below median ocellus (Fig. 1C); F1 hardly longer than wide, as long as F2 (Fig. 2D); clava bi-segmented, sharply pointed apically (Fig. 2D); post-orbital carina joining genal carina at a level of ventral edge of eye (Fig. 3B); pronotal humeral angle sharp, clearly 90° (Fig. 2E); mesonotum densely punctured, bearing relatively long, golden lanceolate setae (Fig. 2E); bottom of punctures on mesonotum and metepimeron and of areola of propodeum granulate (densely reticulate) and dull (Figs 2E, 3B); propodeum with median areola complete, not much longer than adpetiolar areola, distinctly widened posteriorly (1.5× as long as wide) (Fig. 3A); metacoxa 2.5× as long as wide, widened basally (Fig. 1C); metafemur ventrally with 10–12 small teeth following the stout basal one (Fig. 4A, B); tarsal scrobe deep and smooth throughout, reaching sub-basal prominence (Fig. 4B); fore wing bare, only sparse white microtrichiae present on underside (Fig. 3C); STV evidently diverging from anterior margin of wing (Fig. 3C).

###### Description.

***Female*** (holotype, Figs 1–4). Body length: 3.8 mm; fore wing length: 2.3 mm.

*Head* (Fig. 2A−E). Slightly wider than mesoscutum in dorsal view (1.1×), distinctly transverse (1.5× as wide as high in frontal view), and ca. 2.8× as wide as its length in profile. Frontovertex 1.6× as wide as eye height. Vertex almost smooth along OOD, finely densely punctate between lateral ocelli; AOL 0.85× OOL; OOL 1.75× OD; POL 2.14× OOL; discal surface not much expanded, squamosa reticulate, separated from orbit by 5–6 rows of piliferous points, and from median ocellus by three rows; orbital surface transversely alutaceous, laterally with fine upwardly directed long setae; preorbital carina extremely weak; malar area densely finely punctate; malar space 0.78× as long as eye height in lateral view; malar carina absent; suborbital carina distinct; gena coarsely foveolate, with inwardly directed fine setae; post-orbital carina lamellate, joining genal carina at a level of ventral edge of eye, strongly converging to the higher edge of the eye (nearly touching it); occipital area finely densely reticulate (with raised network), with some very superficial punctures in between; occipital carina, just above formen magnum (or dorsally), relatively thick. Interantennal distance moderate (0.6× as wide as torulus diameter); a weak longitudinal carina could be seen between antennal toruli. Frontal lobe very short, not masking clypeus, with free margin entire.

*Antenna* (Fig. 2D). 13-segmented (clava bi-segmented), with sparse short setae; scape relatively long (1.28× as long as eye height), ends just below median ocellus; pedicel cylindrical, 1.65× as long as wide; anellus transverse (0.28× as long as wide); F1 1.1× as long as wide, as long as F2, slightly shorter than F7 (0.78×); clava sharply tapered apically, 2.5× as long as wide. Flagellomeres (except the first) bearing mostly a single row of MPS, two rows for preclaval.

*Mesosoma* (Figs 2E, 3A, B). 1.5× as long as mesoscutum width, with lanceolate setae. Pronotal collar 3.7× as wide as long, finely punctate, with fine sparse setae (setae adpressed and short), its sides slightly convex; lateral panel of pronotum rugose; humeral angle clearly 90°. Mesoscutum 2.7× as long as median length of pronotal collar, setiferous foveolate, the foveolae small anteriorly, with alutaceous interspaces, becoming larger with smooth interspaces posteriorly, widely spaced on scapula leaving smooth areas posteriorly. Notauli distinct, finely crenulate. Tegula broadly angulated posteriorly, smooth to finely alutaceous. Mesoscutellum hardly longer than wide (1.07×), densely setiferous foveolate, foveolae finely reticulate inside, with posterior margin broadly rounded. Axilla almost smooth. Propodeum with median areola distinctly widened posteriorly (1.5× as long as wide), weakly transversely carinate inside, extends to reach adpetiolar areola; prestigmatic areola with lanceolate, rather dense setae. Mesopleuron with adscrobal area coarsely foveolate, foveolae finely punctate inside; femoral depression finely transversely striated, ventral shelf of mesepisternum finely punctate, with adpressed setae. Metepimeron densely, closely foveolate throughout, with fine, adpressed lanceolate setae; metepisternum micro-reticulate, with two median carinae ending on a transverse posterior carina with two large teeth; adpetiolar area concave, with a large irregular projection posteriorly.

*Wings* (Fig. 3C). Fore wing 2.77× as long as wide, bare on upper and undersides; MV 0.68× as long as costal cell; STV slightly longer than wide, forming with anterior margin an angle of ca. 45°. Hind wing bare, with three hamuli.

*Hind leg* (Figs 1C, 4A, B). Metacoxa 2.5× as long as wide, widened basally, slightly shorter than metafemur (0.92×), finely transversely alutaceous on outer-dorsal face, rest densely punctured with short setae more densely distributed basoventrally. Metafemur 1.97× as long as wide, with dense setiferous punctures throughout, outer ventral margin with a stout tooth basally, followed by 12 smaller, similar teeth. Tarsal scrobe long, reaching sub-basal prominence; proximal fourth of metatibia finely punctate; edge of sub-basal prominence with four denticles concealed under white pubescence.

*Metasoma* (Figs 1A−C, 3A). Petiole relatively short (4.5× as long as wide, 0.92× as long as dorsal length of Gt_1_, and 0.68× as long as gaster), with a weak incomplete median carina, extending along its basal half, two (sublateral and lateral) ridges extending along its whole length, area between sublateral ridges faintly coriaceous (smooth apically). Gaster subcircular in lateral view (1.45× as long as height), somewhat ovoid in dorsal view. Gt_1_ long, occupying most of gaster (0.75× as long as the whole length of gaster in dorsal view), deeply concave posteriorly, mostly smooth (densely finely punctulate postero-laterally); remaining tergites short, densely finely punctulate, finely setose. Gt_2_ slightly concave posteriorly. Ovipositor slightly extended to apex of gaster.

*Color* (Figs 1A−C, 3C). Head including antennal flagellomeres and clava are black, except a broad band around inner margin of eye, malar area, clypeus and antennal scape to anellus are reddish brown. Mesosoma including legs and metasoma reddish brown, except anterior third of mesoscutal middle lobe, antero-inner corner of scapula, posterior margin of mesoscutellum, dorsellum, most of propodeum and ovipositor are black; propodeum postero-laterally reddish brown; outer faces of fore and mid femora and tibiae, dorsal face of metacoxa, inner face of metafemur, basal two-thirds of petiole and Gt_1_ dorsally with blackish tint. Tegula glassy yellowish red. Wings hyaline, with pale brown to yellowish veins.

***Male*** (**Paratype**, Fig. 5A−C). Differs from the female in the following: AOL slightly longer than OOL (1.16×); OOL 1.2× as long as OD; POL 2.8× as long as OOL; interantennal distance 1.2× as long as antennal torulus diameter; F1 longer (1.4× as long as wide, 1.06 as long as F7); mesoscutum length 3.3× as long as pronotum median length; metacoxa shorter, ca. 1.18× as long as width; petiole longer (5.7× as long as wide), with medial carina extending along its whole length; head and mesosoma completely black (except clypeus and tegula); metacoxa and petiole mostly black.

###### Remarks.

*Epitranus delvarei* differs from all species of the genus in having small teeth on the metafemur; the presence of dense reticulation in the bottom of punctures on mesoscutellum, metepimeron, as well as areola of propodeum; tarsal scrobe of metatibia reaching sub-basal prominence.

###### Hosts.

Unknown.

###### Distribution.

Saudi Arabia (Al-Baha and Asir regions).

###### Etymology.

The new species is named *delvarei*, in honor of Gerard Delvare, for his kind efforts and help in the identification of several chalcid species.

##### Epitranus similis

Taxon classificationAnimaliaHymenopteraChalcididae

Gadallah & Soliman sp. nov.

0C0687C8-CEE8-524E-810C-85BE7763F7B1

http://zoobank.org/A190D08C-92B7-4662-9660-B1C642740266

**Figure 6.**
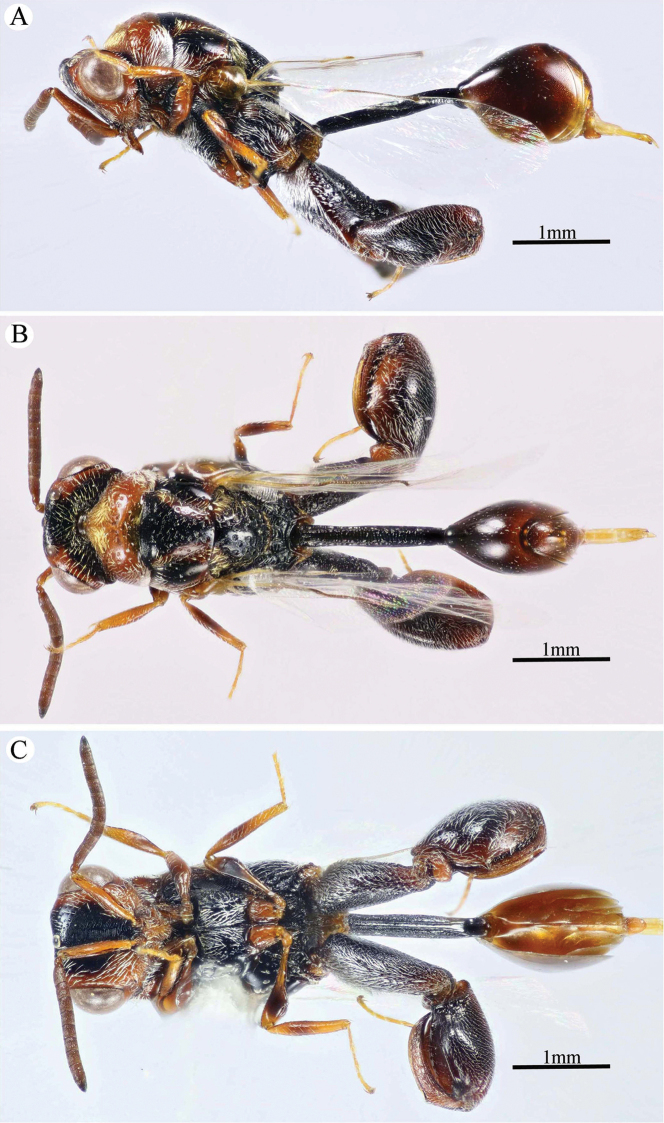
*Epitranus similis* Gadallah & Soliman, sp. nov. (holotype male) **A, B, C** habitus (lateral, dorsal, and ventral views respectively).

**Figure 7.**
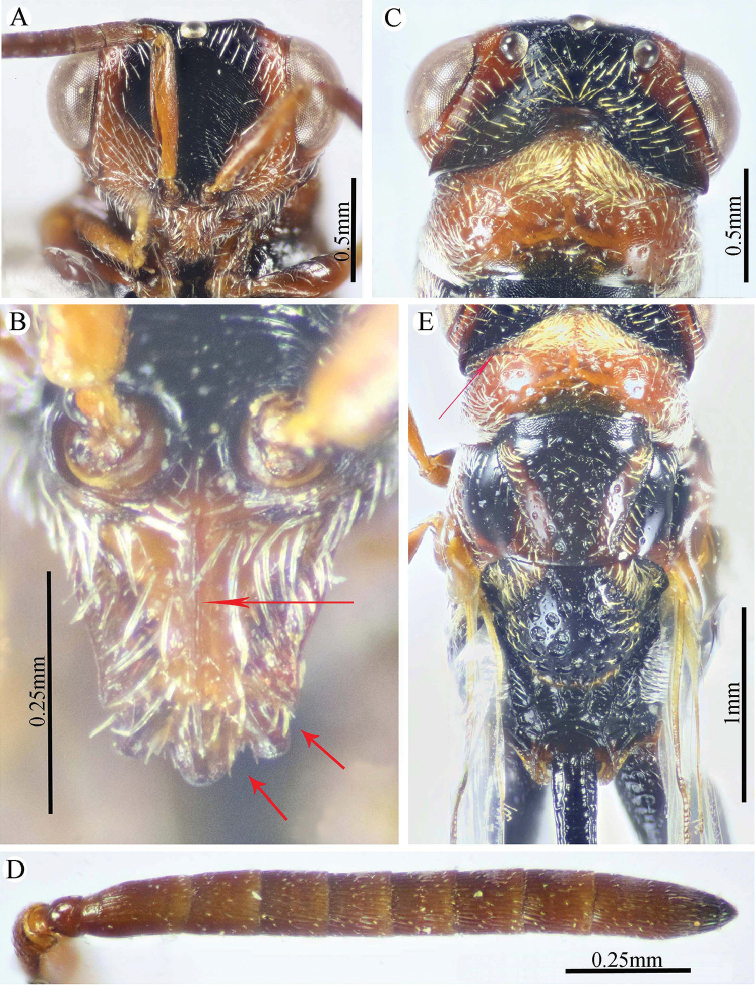
*Epitranus similis* Gadallah & Soliman, sp. nov. (holotype male) **A** head (frontal view) **B** lower part of face showing frontal lobe (frontal view) **C** head & pronotum (dorsal view) **D** antennal pedicel and flagellum **E** mesosoma and part of gastral petiole (dorsal view).

**Figure 8.**
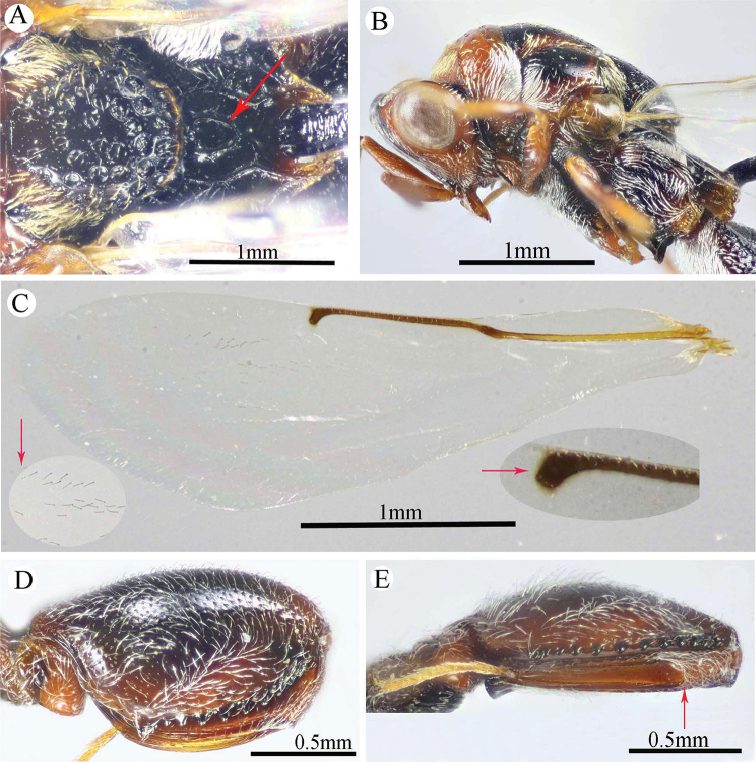
*Epitranus similis* Gadallah & Soliman, sp. nov. (holotype male) **A** mesoscutellum, metanotum & propodeum (dorsal view) **B** head & mesosoma (lateral view) **C** fore wing (parts of wing membrane and MV and STV magnified) **D, E** hind leg, excluding coxa (outer and ventral views respectively).

**Figure 9.**
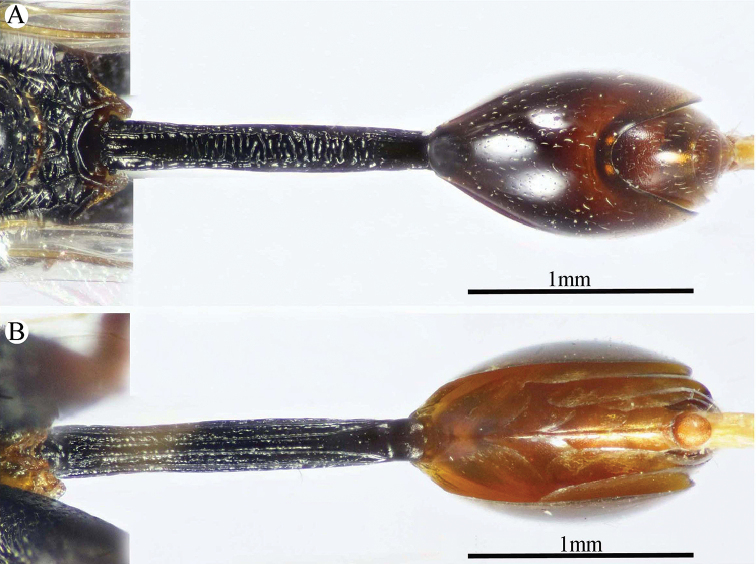
*Epitranus similis* Gadallah & Soliman, sp. nov. (holotype male) **A, B** metasoma (dorsal and ventral views respectively).

Figures 6 , 7 , 8 , 9

###### Type material.

***Holotype*** ♂. **Kingdom of Saudi Arabia**, Asir, Abha, Garf Raydah Natural Reserve [18°11'35.74"N, 42°23'30.24"E, Alt. 1805 m], sweeping net, 5.IX.2015, leg. Ahmed M. Soliman [KSMA]; ***Paratypes***: 2♂, **Kingdom of Saudi Arabia**, same data as for holotype [KSMA].

###### Diagnosis.

Frontal lobe distinctly long, sub-trapezoidal in shape as its sides are straight and regularly converging ventrally, with a longitudinal median carina extending on its whole length, and free margin with three lobes (outer lobes notched subapically) (Fig. 7B); OOL 0.80–0.85× as long as OD, and ca. 0.5× as long as AOL (Fig. 7C); POL ca. 4.3× as long as OOL (Fig. 7C); F1 moderately long, ca. 1.7× as long as wide (Fig. 7D); pronotal collar laterally, scapula anterolaterally, propodeum on prestigmatic areola, mesepisternum, metepimeron dorsally and basoventral surface of metacoxa densely clothed with whitish setae masking integument beneath (Figs 6A, C, 8B); pronotal collum with dense, long golden setae on two submedian patches (Fig. 7C); propodeal median areola deep, with lateral ridges converging posteriorly to meet before the adpetiolar areola (Y-like raised carina) (Fig. 8A); metafemur toothed ventrally, with nine or ten spaced teeth following the stout sub-basal tooth (Fig. 8D, E); tarsal groove of metatibia fully occupying the completely delimited smooth and deep metatibial process, reaching the sub-basal prominence anteriorly (Fig. 8E); petiole very long, 7.12–7.65× as long as wide (Fig. 9A); gaster relatively short (1.25–1.40× as long as height in profile) (Fig. 6A).

###### Description.

***Male*** (**holotype**). Body length 5.5 mm. fore wing length 3.3 mm.

*Head* (Figs 7A−D, 8B). Triangular in frontal view, 1.25× as wide as high, wider than mesoscutum in dorsal view (1.2×), 2.6× as wide as its length in profile. Frontovertex 1.5× as wide as eye height; AOL 2.0× OOL; OOL 0.85× OD; POL 4.3× OOL; supra antennal surface absent; frons transversely finely strigulate medially beneath antennal scape (at scrobe), laterally with sparse setiferous punctures, the setae lanceolate and long; preorbital carina absent; malar area mostly polished, with scattered superficial setiferous punctures; malar space ca. 0.6× as long eye height in profile; malar carina absent; gena broad, nearly smooth, with a row of setae directed inwards along post-orbital carina that is well-developed, lamellate and joining genal carina at a level of ventral edge of eye; post-orbital carina hardly converging to the higher edge of the eye (nearly parallel); suborbital carina weak. Occiput alutaceous, with sparse setiferous punctures (setae long and dispersed, pale yellow). Interantennal projection absent; interantennal distance ca. 0.5× as long as torulus diameter; frontal lobe quite long, with subantennal distance 4.0× as long as interantennal distance, ventral margin with two pairs of indentations delimiting three lobes, the outer ones are narrowly rounded; projection with a sharp longitudinal median carina extended throughout its length; surface of projection with long, lanceolate and whitish setae.

*Antenna* (Fig. 7D). Scape 1.35× as long as eye height, ending very closely to median ocellus, its ventral face strongly excavated, the excavation densely and finely pubescent; pedicel hardly longer than wide (1.15×); anellus transverse (0.4× as long as wide); F1 moderate, 1.7× as long as wide, 1.3× as long as F2 and F7 as well; clava 2.35× as long as wide.

*Mesosoma* (Figs 7C, E, 8A, B). 1.65× as long as mesoscutum width. Pronotal collar 2.45× as wide as long, mostly smooth on disc, with scattered punctures bearing fine setae; collar laterally and collum densely setiferous punctulate, the setae forming tufts that are inwardly oriented, white on the former and golden yellow on the latter; pronotal sides slightly convex; lateral carinae sharp and extending dorsally, not meeting medially; humeral angle rounded; pronotal lateral panel finely alutaceous, shiny. Mesoscutum 2.25× as long as median length of pronotum, middle lobe finely alutaceous anteriorly, followed by deep and sparse setiferous punctures bearing fine setae; scapula nearly smooth, with few scattered punctures, antero-laterally clothed with dense lanceolate whitish setae; notauli deep, foveolate; axilla with dense, upwardly directed whitish setae, integument smooth beneath; tegula large, depressed near posterior margin, smooth anteriorly, finely alutaceous posteriorly and laterally, with broad angulate posterior margin that distinctly overlap base of hind wing. Mesoscutellum convex, hardly longer than wide (1.1×), irregularly deeply foveate, the foveae large, widely separated medially and closer laterally, posterior margin broadly rounded, strigose. Propodeum deeply areolate; median areola 1.37× as long as wide, with lateral ridges converging posteriorly and meeting slightly before adpetiolar areola; lateral areola transversely carinate; prestigmatic areola densely setose, setae oriented inwards. Adscrobal area of mesopleuron densely clothed with long, suberect and whitish setae, femoral scrobe finely transversely strigose, ventral shelf of mesepisternum sparsely punctate, with adpressed long setae, interspaces between punctures smooth; epicnemial carina lamellate. Metepimeron closely foveolate throughout (bottom of foveae smooth), with dense, long adpressed and whitish setae; metepisternum densely reticulate, with two median carinae diverging posteriorly followed by two large and sharp submedian teeth; subcoxal teeth small; adpetiolar area concave, longitudinally striated, with a median longitudinal carina ends posteriorly with a strong subpentagonal subpetiolar areola.

*Wings* (Fig. 8C). Fore wing 3.2× as long as wide, bare on upper side, sparsely setose subapically on underside; MV ca. 0.72× as long as costal cell; STV rudimentary (1.25× as long as wide), strongly diverging from anterior margin of wing at an angle of ca. 80°. Hind wing hyaline and asetose, with three hamuli.

*Hind leg* (Figs 6C, 8D, E). Metacoxa widened basally, 2.45× as long as wide, 0.9× as long as metafemur, densely setose on ventral side, punctured but with transverse ridges near apex on outer dorsal side. Metafemur 1.9× as long as wide, with dense setiferous punctures on outer face, its ventral margin with a triangular sub-basal tooth followed by nine teeth that are equally separated, progressively smaller towards apex. Tarsal scrobe of metatibia fully occupying the completely delimited, smooth and deep metatibial process, reaching sub-basal prominence anteriorly (Fig. 12E), the edge of the later with four denticles concealed by pubescence (could be seen when examined from dorsal view).

*Metasoma* (Figs 6A, 9A, B). Petiole quite long, 7.12× as long as broad, 1.58× as long as length of Gt_1_ in dorsal view, and 1.2× as long as length of gaster in dorsal view, dorsally with two, lateral and sublateral, longitudinal ridges that is vague along the apical two-thirds, the area between sublateral ridges transversely wrinkled. Gaster ovoid in dorsal view, 1.25× as long as its height in lateral view. Gt_1_ long (0.75× as long as gaster in dorsal view), deeply concave posteriorly, sparsely finely setiferous punctate (setae fine and short, punctures dense postero-laterally); remaining tergites short, sparsely finely punctate and finely setose.

*Color* (Figs 6A−C, 8C). Body black, except the following parts, bright reddish brown: frontal lobe, malar area, a relatively broad strip around eye, gena, pronotum, a lateral longitudinal strip on middle lobe of mesoscutum, lateral and posterior borders and postero-inner corner of scapula, inner part of axilla, area around epicnemial carina on mesopleuron, upper part of metepimeron, and posterior part of adpetiolar area on metepisternum. Gaster reddish brown with black tint dorsally. Antennal scape and pedicel reddish brown, flagellum brown. Legs reddish brown, with black tint on mesofemur, ventral face of metacoxa and outer face of metafemur. Tegula glassy golden yellow. Wings hyaline with brown veins that are paler on hind wing. Genitalia pale yellow.

***Female*.** Unknown.

###### Variation.

The paratype specimens differ from the holotype specimen in the predominance of red brown color on: head (except post-orbital and occipital carinae in one of the paratype specimens or a band along occipital carina, post-orbital carina and a narrow longitudinal median strip on the frons in the other paratype specimen); middle lobe of mesoscutum (except a triangular area on disc); lateral lobe of mesoscutum (except an oval area on disc); the whole axilla, mesoscutellum (except longitudinal median strip); the whole metapleuron; metacoxa and metafemur (except black tint on the former).

###### Remarks.

The new species is morphologically similar to *E. nitidus* (Schmitz) (Democratic Republic of Congo) especially the identical frontal projection; the absence of interantennal projection; similar flagellum; the presence of outstanding setae on mesosoma; similar STV, and similar petiole. But differs from it by the partly reddish head and mesosoma (entirely black in *E. nitidus*); the presence of distinctive setation on different parts of mesosoma as reported above (mesosoma with regular setation in *E. nitidus*); propodeum with petiolate median areola (complete in *E. nitidus*); shorter and relatively stouter metacoxa (quite slender in *E. nitidus*).

###### Etymology.

The word *similis* is an adjective in Latin and means similar or resembling, referring to the similarity of this species to *E. nitidus*.

###### Hosts.

Unknown.

###### Distribution.

Saudi Arabia (Asir region).

##### Epitranus subinops

Taxon classificationAnimaliaHymenopteraChalcididae

Soliman & Gadallah sp. nov.

DD26AA7B-C9EC-57F9-87B1-886FF594E0B7

http://zoobank.org/EDF024B1-4346-44E6-A08E-DAC73BC1CCB8

**Figure 10.**
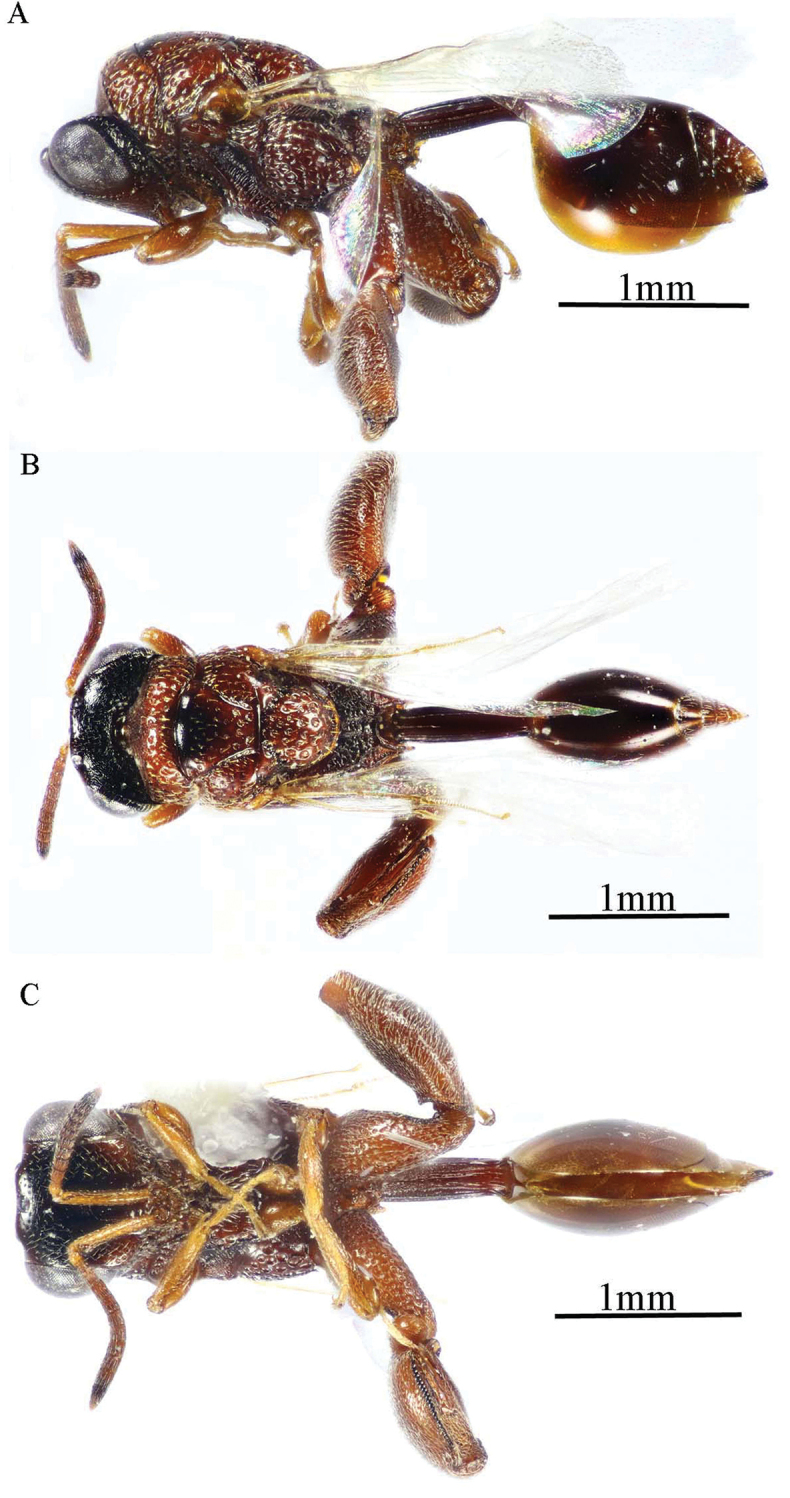
*Epitranus subinops* Soliman & Gadallah, sp. nov. (holotype female) **A, B, C** habitus (lateral, dorsal and ventral views respectively).

**Figure 11.**
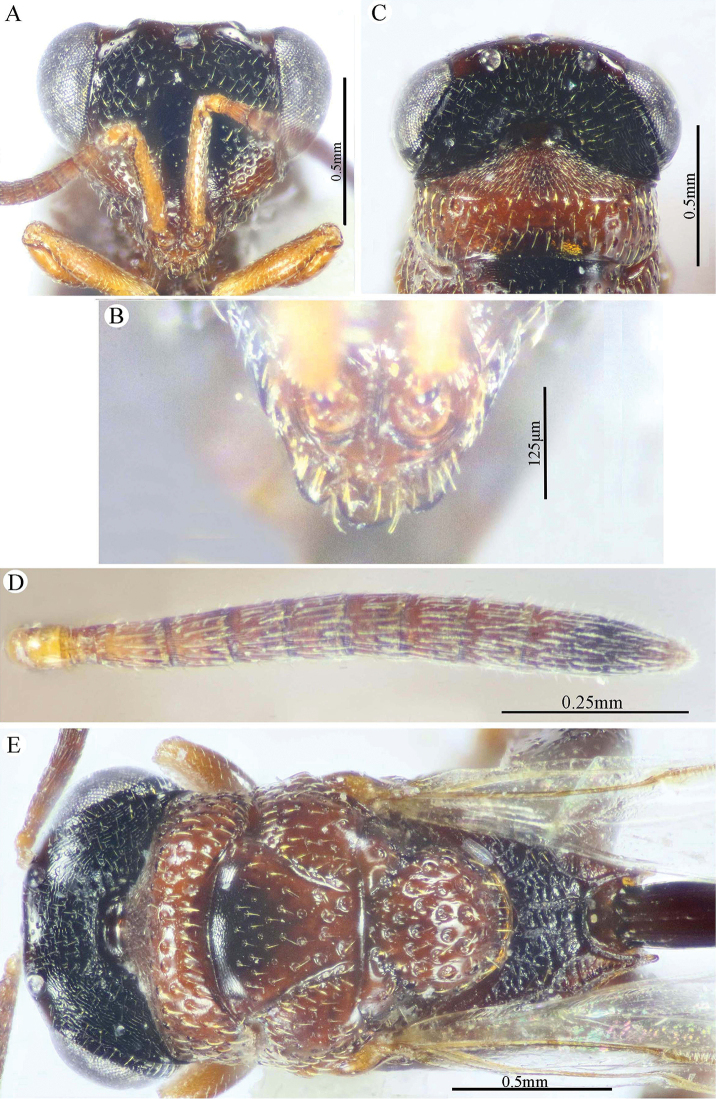
*Epitranus subinops* Soliman & Gadallah, sp. nov. (holotype female) **A** head (frontal view) **B** lower part of face showing frontal lobe (frontal view) **C** head and pronotum (dorsal view) **D** antennal pedicel and flagellum **E** head and mesosoma (dorsal view).

**Figure 12.**
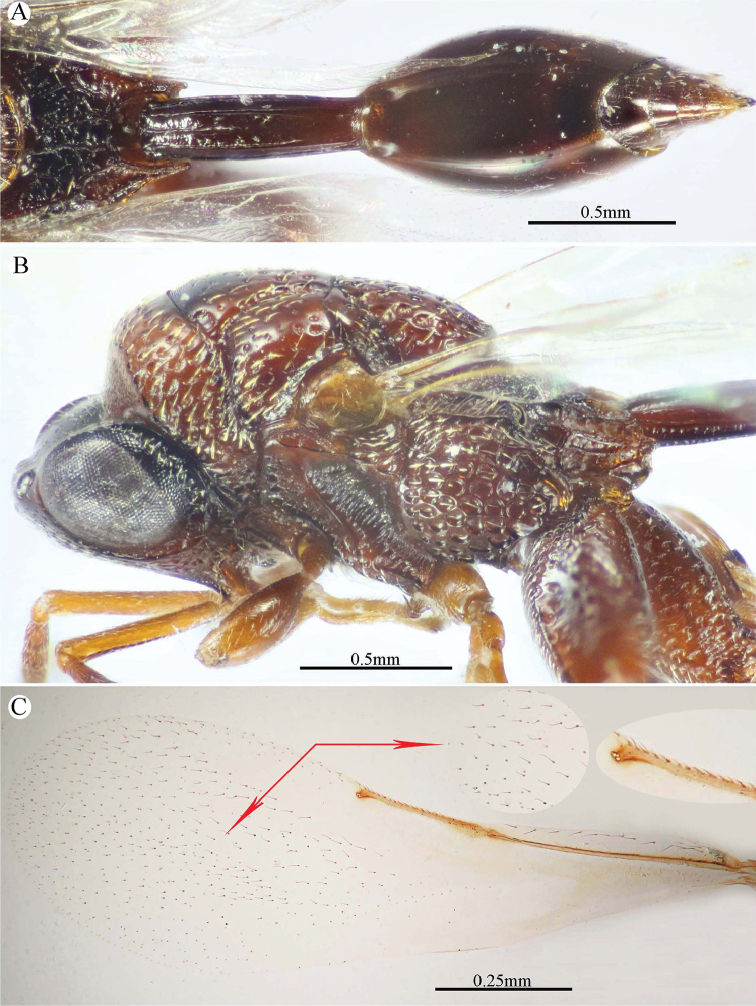
*Epitranus subinops* Soliman & Gadallah, sp. nov. (holotype female) **A** propodeum and metasoma (dorsal view) **B** head including antennal scape and mesosoma (lateral view) **D** fore wing (parts of wing membrane and MV and STV magnified).

**Figure 13.**
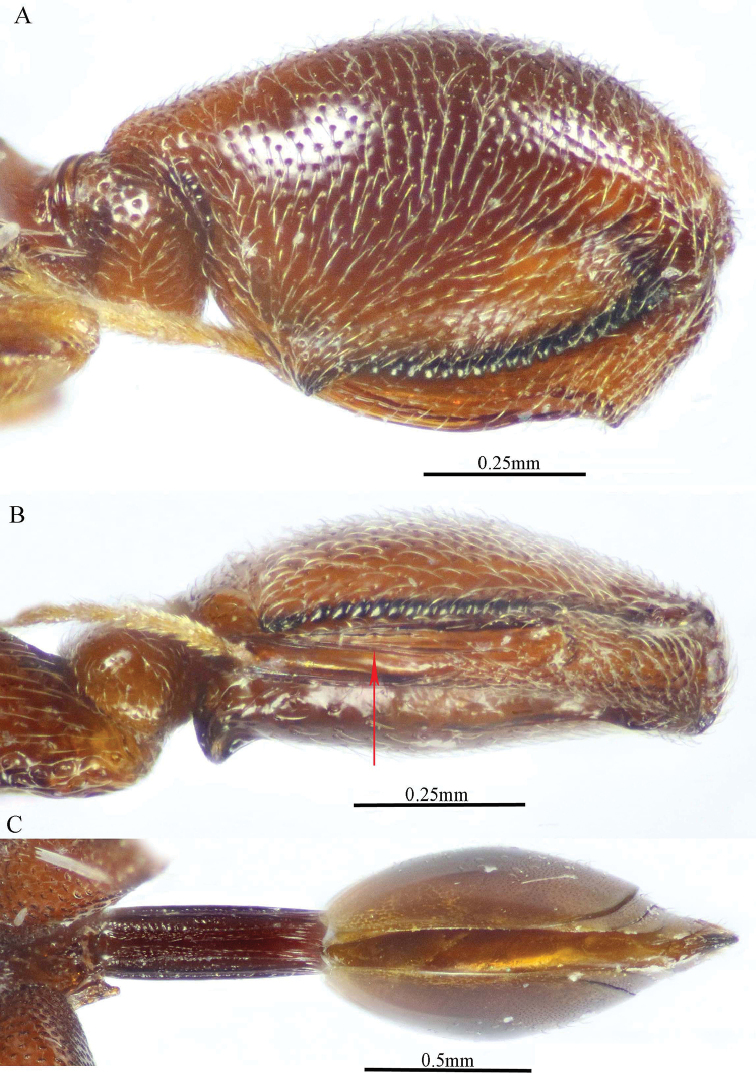
*Epitranus subinops* Soliman & Gadallah, sp. nov. (holotype female) **A, B** hind leg, excluding coxa (outer and ventral views respectively) **C** metasoma (ventral view).

Figures 10 , 11 , 12 , 13

###### Type material.

***Holotype*** ♀: **Kingdom of Saudi Arabia**, Asir, Regal Alma, Wadi Kasan (2 km North of El-Hebeal) [18°6'59.89"N, 42°13'54.92"E, Alt. 487 m], sweeping net, 12.IV.2019, leg. Ahmed M. Soliman [KSMA].

###### Diagnosis.

Frontal lobe relatively long, its free margin trilobate (Fig. 11B); OOL ca. 1.5× as long as OD, and as long as AOL (Fig. 11C); POL 2.4× as long as OOL (Fig. 11C); interantennal projection well developed (lamellate) (Fig. 11B); scape ends a long distance from median ocellus (Fig. 11A); F1 relatively long, 1.75× as long as wide, following flagellomeres shorter, subequal (Fig. 11D); clava bi-segmented, relatively long ca. 2.7× as long as wide, tapering apically (Fig. 11D); frons with supra antennal surface delimited by step-like margin; frons sparsely punctured, with fine setae directed upwards, integument smooth behind (Fig. 11A); post-orbital carina joining genal carina at a level distinctly above the ventral edge of the eye (Fig. 12B); pronotal humeral angle rather sharp (Fig. 11C); mesoscutum with short setae and very sparse small punctures on anterior third of middle lobe (Fig. 11E); bottom of punctures on mesonotum and metepimeron smooth (Figs 11E, 12B); propodeum densely areolate, median areola complete (Fig. 12A); metafemur serrulate ventrally following a stout tooth at base (Fig. 13A, B); metatibia with oblique carina inside metatibial process (Fig. 13B); tarsal scrobe almost reaching sub-basal prominence (Fig. 13B); fore wing densely setose along apical two thirds (Fig. 12C); STV present but reduced, 2.0× as long as wide (Fig. 12C).

###### Description.

***Female*** (holotype). Body length 3.4–3.9 mm. Fore wing length 2.1–2.5 mm.

*Head* (Figs 11A−C, 12B). Transverse (1.16× as wide as high in frontal view), slightly wider than mesoscutum in dorsal view (1.1×), and ca. 2.45× as wide as its length in profile. Frontovertex 1.25× as wide as eye height. Vertex almost smooth, sparsely punctate between median ocellus and eyes, with AOL as long as OOL; OOL 1.5× OD; POL 2.4× OOL; orbital surface superficially transversely alutaceous, laterally with fine sparse setae directed upwards; malar area superficially wrinkled; malar space 0.57× as long as eye height in lateral view; malar carina faint and polished; gena coarsely foveolate, nearly bare; post-orbital carina well developed, joined genal carina at a level distinctly above the ventral edge of the eye, distinctly converging to the higher edge of the eye; preorbital and suborbital carinae developed. Occipital area densely reticulate; interantennal distance distinctly short, 0.4× as long as torulus diameter, interantennal projection well developed (lamellate); frontal lobe relatively long (subantennal distance 3.3× as long as interantennal distance), free margin with three lobes.

*Antenna* (Fig. 11D). 13-segmented, clava bi-segmented, with few scattered short setae; scape moderately long, longer than eye height (1.33×), ending a distance before median ocellus, densely punctured throughout; pedicel relatively short, conical shape, approximately as long as its width; anellus transverse, ca. 0.6× as long as wide; F1 relatively long, 1.75× as long as wide, following funiculars distinctly shorter, subequal; clava ca. 2.7× as long as wide, tapered apically.

*Mesosoma* (Figs 11E, 12A, B). 1.8× as long as mesoscutum width, with relatively short setae, that are somewhat thickened on pronotum and mesoscutellum. Pronotal collar 3.0× as wide as long, sparsely setiferous foveolate, that are denser laterally, with sides slightly convex; humeral angle sharp, nearly 90°; lateral carina not extended dorsally on collar. Mesoscutum 2.9× as long as pronotal collar median length, sparsely finely punctulate on anterior half of middle lobe, its anterior margin finely alutaceous; posterior half of middle lobe with large irregular foveolae, lateral lobes with dense setiferous punctures. Notauli very distinct and deep, linear (not crenulate). Tegula broadly rounded posteriorly, smooth. Mesoscutellum slightly longer than wide (1.1×), setiferous foveolate, foveolae smooth on bottom, with posterior margin broadly rounded. Propodeum strongly areolate, median areola slightly widened posteriorly, 2.6× as long as wide, weakly transversely striated on bottom, its lateral carinae slightly diverging posteriorly and reaching transverse carina of adpetiolar areola; submedian and basolateral areolae fused. Mesopleuron with adscrobal area coarsely foveolate, foveolae finely punctate inside; femoral scrobe coarsely transversely ridged, ventral shelf of mesepisternum finely punctate, with adpressed setae. Metepimeron densely, closely foveolate throughout, with fine, adpressed setae; metepisternum largely areolate throughout (bottom of areolae densely reticulate), with two median carinae slightly diverging posteriorly followed by two large and sharp submedian teeth; adpetiolar area concave, nearly smooth, with a median longitudinal carina ends posteriorly with a strong subpentagonal areola.

*Wings* (Fig. 12C). Fore wing ca. 3.0× as long as wide, rather densely setose on the underside of apical two-thirds, setae distinctly long; MV 0.6× as long as costal cell; STV somewhat reduced (0.1× as long as MV), 2.0× as long as wide, forming with anterior margin an angle of ca. 45°. Hind wing sparsely setose apically, with three hamuli.

*Hind leg* (Figs 10C, 13A, B). Metacoxa 2.2× as long as wide, widened basally, slightly shorter than metafemur (0.9×), finely transversely alutaceous on outer dorsal face, rest densely setiferous punctulate, interspaces between punctures smooth. Metafemur 1.75× as long as wide, with dense setiferous punctures throughout, outer ventral margin with broad triangular tooth basally, followed by a serrulation of minute teeth. Metatibia with an oblique carina inside metatibial process; tarsal scrobe almost reaching sub-basal prominence; sub-basal prominence is formed from three small blunt teeth partly hidden by dense pubescence.

*Metasoma* (Figs 10A, B, 12A, 13C). Petiole relatively short, 3.5× as long as wide, 0.92× as long as dorsal length of Gt_1_, and ca. 0.6× as long as gaster, with an incomplete median carina (0.45× as long as petiole length), two incomplete submedian carinae (0.73× as long as petiole length), and two complete lateral ridge, area between sublateral ridges nearly smooth and shiny. Gaster fusiform in dorsal view, 1.55× as long as its height in profile. Gt_1_ 0.6× as long as the whole length of gaster in dorsal view, deeply concave posteriorly, almost entirely smooth; remaining tergites short, densely finely punctate at base, finely setose. Gt_2_ slightly concave posteriorly. Ovipositor slightly extended behind gaster.

*Color* (Figs 10A−C, 12C). Body generally reddish to reddish brown, with the following parts black: head (except lateral sides just below lower edge of eyes, and frontal lobe), anterior margin of mesoscutum middle lobe, propodeum (except postero-lateral margins). Metasoma dark reddish brown; antenna with scape and pedicel bright red, rest reddish brown; tegula testaceous. Wings hyaline with yellowish brown veins that are paler on hind wing.

###### Remarks.

The new species closely similar to *E. inops*, but differs in the following: metatibia with oblique carina inside metatibial process (Fig. 13B) (metatibia without such carina in *E. inops* (Fig. 27C)); tarsal scrobe almost reaching sub-basal prominence (Fig. 13B) (tarsal scrobe short, far from reaching sub-basal prominence in *E. inops* (Fig. 27C)); frons with supra antennal surface completely delimited by a step-like margin (Fig. 11A) (supra antennal surface delimited only laterally by faint step-like ridge in *E. inops* (Fig. 26A)); mesoscutum with short, very small and sparse punctures on anterior part of middle lobe, while posterior area with coarse irregular foveolation (Fig. 11E) (setae on mesoscutum longer, denser and coarser on the whole middle lobe in *E. inops* (Fig. 25B)); mesoscutellum convex when seen in profile (Fig. 10A) (flat in *E. inops* (Fig. 25A)).

***Male***. Unknown.

###### Etymology.

The new species name *subinops* refers to the similarity of this species to *E. inops*.

###### Hosts.

Unknown.

###### Distribution.

Saudi Arabia (Asir region).

#### List of new records

##### Epitranus clavatus

Taxon classificationAnimaliaHymenopteraChalcididae

(Fabricius, 1804)

729A41E2-91BD-5E2F-975C-6F5148544126

**Figure 14.**
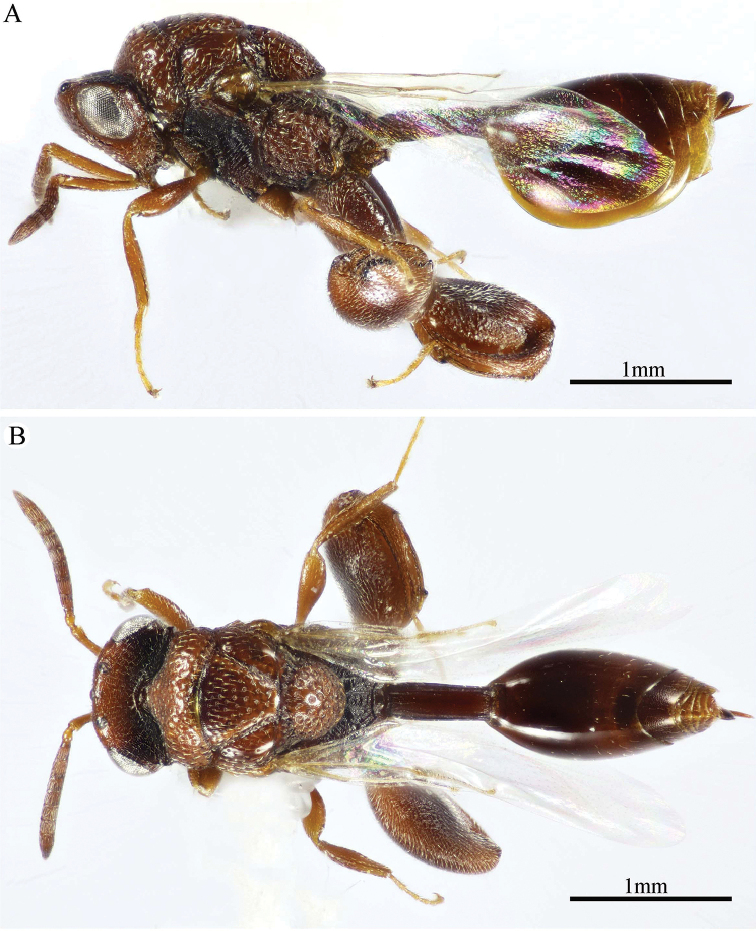
*Epitranus clavatus* (Fabricius) (female) **A, B** habitus (lateral and dorsal views respectively).

**Figure 15.**
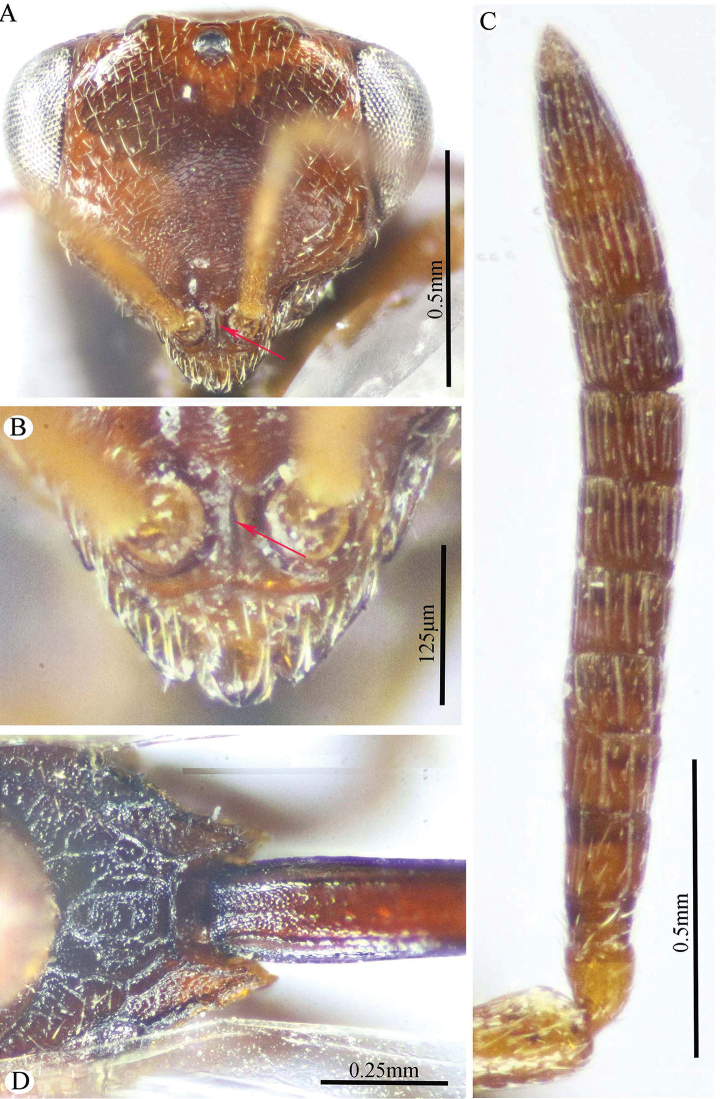
*Epitranus clavatus* (Fabricius) (female) **A** head (frontal view) **B** lower part of face showing frontal lobe (frontal view) **C** antennal pedicel and flagellum **D** propodeum and part of metasomal petiole (dorsal view).

**Figure 16.**
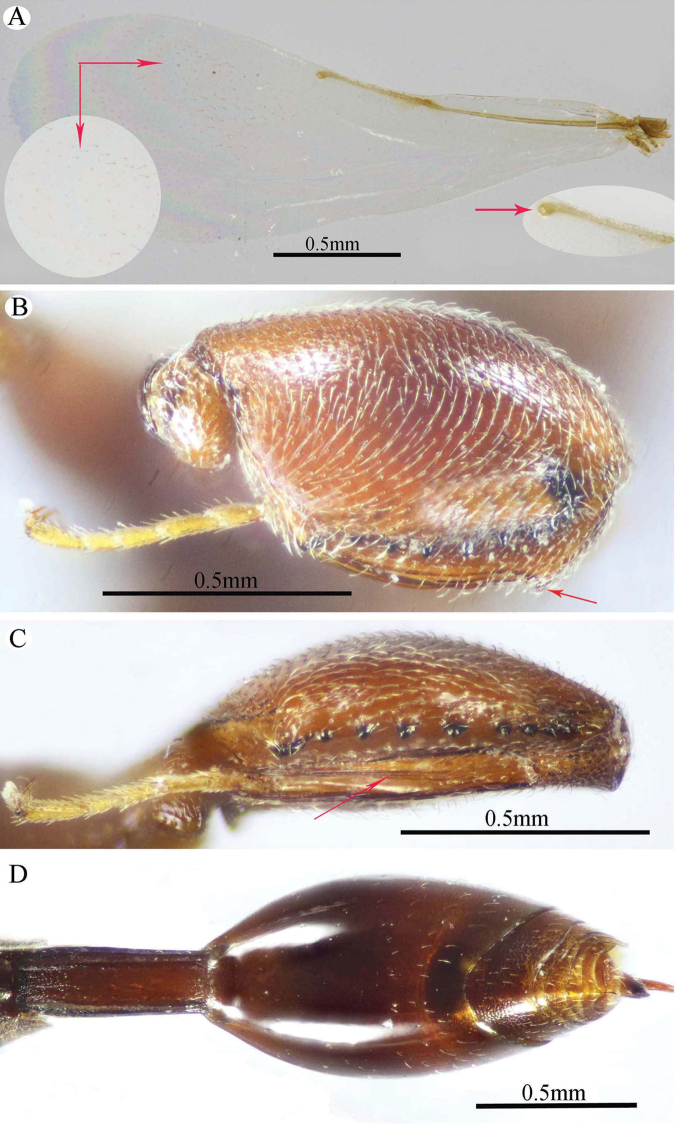
*Epitranus clavatus* (Fabricius) (female) **A** fore wing (parts of wing membrane and MV and STV magnified) **B, C** hind leg, excluding coxa (outer and ventral views respectively) **D** metasoma (dorsal view).

Figures 14 , 15 , 16

Chalcis clavata Fabricius, 1804: 162; Bouček, 1982: 594: lectotype designation.
Epitranus fulvescens Walker, 1834: 26–27; Bouček, 1982: 594: synonymy.
Epitranus lacteipennis Cameron, 1883: 187–188; Bouček, 1982: 594: synonymy.
Anacryptus insidiosus Masi, 1917: 129–130; Bouček, 1982: 594: synonymy.
Anacryptus anpingius Masi, 1933: 14–15; Bouček, 1982: 594: synonymy.
Anacryptus cawnporensis Mani & Dubey in Mani, Dubey, Kaul & Saraswat, 1973: 30–31; Bouček, 1982: 594: synonymy.
Epitranus clavatus (Fabricius): Bouček, 1982: 594.

###### Re-description.

***Female*** (Figs 14–16). Body length ca. 3.75 mm. Fore wing length ca. 2.5 mm. Head and mesosoma mostly reddish, the later variously maculated with black (Fig. 14A, B), tegula brownish testaceous (Fig. 14B). This species is recognized by the following combination of characters: frontal lobe relatively long, its ventral margin with two submedian indentations (Fig. 15B); subantennal distance ca. 1.7× as long as interantennal distance, without median longitudinal carina; subtorular carina present; interantennal projection as small lamina (Fig. 15B); post-orbital groove granulate; post-orbital carina joining genal carina at a level with ventral edge of eye (Fig. 14A); outline of frons slightly and regularly convex in dorsal view; supra antennal surface hardly delimited laterally by very faint step-like ridge; discal area very faintly strigulate, separated from inner orbit and median ocellus by four or five rows of setiferous points (Fig. 15A); flagellum somewhat slender (Fig. 15C), 0.82× as long as head width; funiculars all somewhat longer than wide; mesosoma convex, its dorsal outline evidently so (Fig. 15C); pronotal collar rounded anteriorly on dorsum (Fig. 14A); interspaces on mesonotum very faintly alutaceous; interspaces on mesepisternum and metepimeron coriaceous, dull (Fig. 14A); surface of propodeum densely reticulate, median areola complete but delimited by very faint submedian carina, sublateral carinae well raised on joining lateral carinae of adpetiolar areola, the latter nearly truncate anteriorly (Fig. 15D); pronotum, scapula (Fig. 14A, B) and ventral face of metacoxa sparsely setose, setae distinctly fine and short especially on pronotal collum; occiput (Fig. 14B) and propodeal prestigmatic areola (Fig. 15D) nearly bare, with scattered short and fine setae, the former finely alutaceous beneath. Fore wing (Fig. 16A) with sparse setae and microtrichiae on apical half of underside; STV distinctly oblique forming with the anterior margin of the wing an angle of ca. 35°. Metafemur with a stout basal tooth basoventrally, followed by eight small, widely spaced teeth (Fig. 16B); metatibial process with oblique carina inside, isolating the tarsal scrobe on inner side of tibia, the scrobe nearly reaching the sub-basal prominence anteriorly (Fig. 16C); prominence with three or four denticles concealed by the pubescence (when examined from behind). Metasomal petiole short, 2.7× as long as wide, 0.7× as long as dorsal length of Gt_1_, 0.5× as long as gaster (Fig. 16D), slightly swollen sub-basally, with two (sublateral and lateral) ridges extending along its whole length, area between sublateral ridges flat and faintly finely punctate. Gaster relatively elongate (1.5× as long as high).

***Male.*** Similar to female but differs in having: body with extensive black tint on different parts; flagellum longer and slenderer (1.13× as long as head width); metasomal petiole longer (4.1× as long as wide, ca. 0.66× as long as dorsal length of gaster), with sides parallel and dorsum with weak median carina.

###### Hosts.

Small Lepidoptera such as fungus moths (Tineidae): *Tinea antricola* Meyrick, and *Crypsithyris* sp. (Bouček 1982, Noyes 2019).

###### Material examined.

1♀&1♂, Kingdom of Saudi Arabia, Asir, Abha, Garf Raydah Natural Reserve [18°11'40.98"N, 42°23'45.66"E, Alt. 1861 m], sweeping net, 12.IV.2019, leg. Ahmed M. Soliman [KSMA]; 1♂, Kingdom of Saudi Arabia, Asir, Abha, Wadi Marabah [18°10'09.59"N, 42°22'15.12"E, 1205 m], sweeping net, 13.IV.2019, leg. Ahmed M. Soliman [KSMA].

###### Distribution.

This species probably originates from SE Asia and was repeatedly introduced following trading (Bouček 1982), Iran (Moravvej et al. 2018), Saudi Arabia (Asir region) (new record).

##### Epitranus hamoni

Taxon classificationAnimaliaHymenopteraChalcididae

complex

DA82EBAB-489F-5A15-ADDF-9B192C1BB974

Figures 17 , 18 , 19 , 20 , 21 , 22 , 23 , 24

Spilochalcis hamoni Risbec, 1957: 240.

###### Diagnosis.

**Figure 17.**
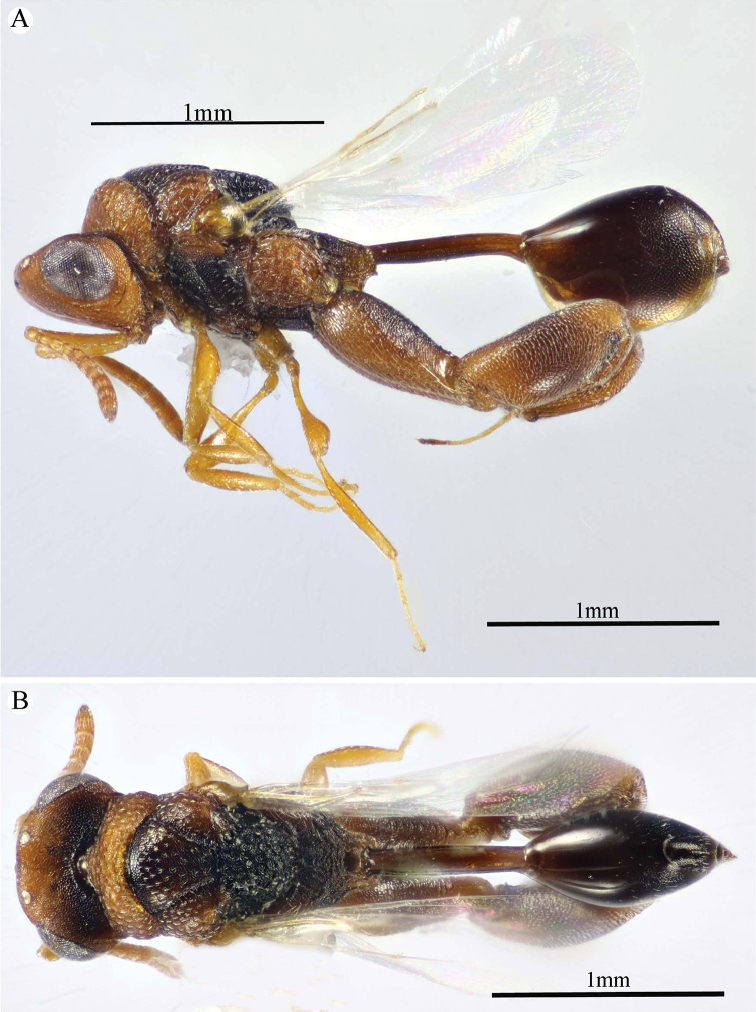
*Epitranus hamoni* complex (female, dark form) **A, B** habitus (lateral and dorsal views respectively).

**Figure 18.**
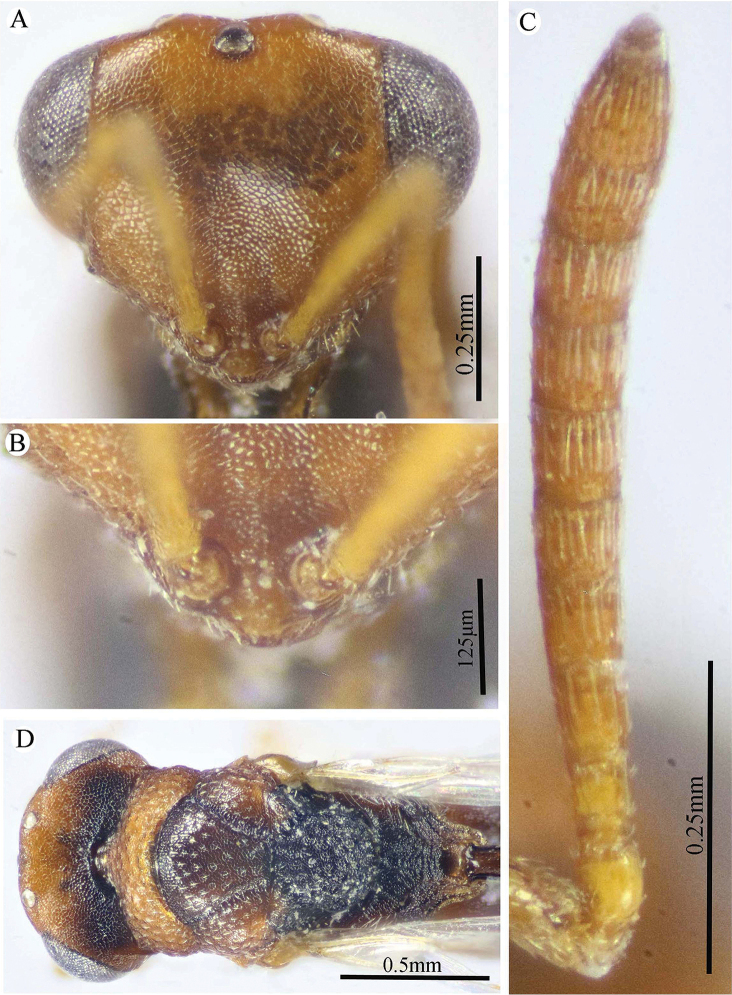
*Epitranus hamoni* complex (female, dark form) **A** head (frontal view) **B** lower part of face (frontal view) **C** antennal pedicel and flagellum **D** head and mesosoma (dorsal view).

**Figure 19.**
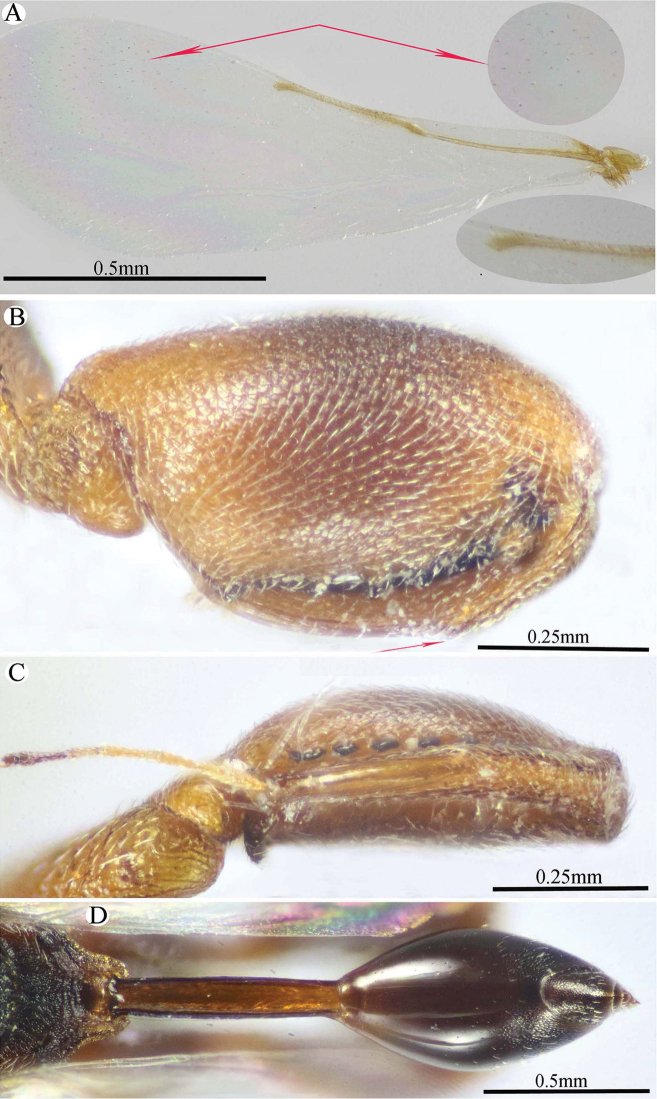
*Epitranus hamoni* complex (female, dark form) **A** fore wing (parts of wing membrane and MV and STV magnified) **B, C** hind leg, excluding coxa (outer and ventral views respectively) **D** propodeum and metasoma (dorsal view).

**Figure 20.**
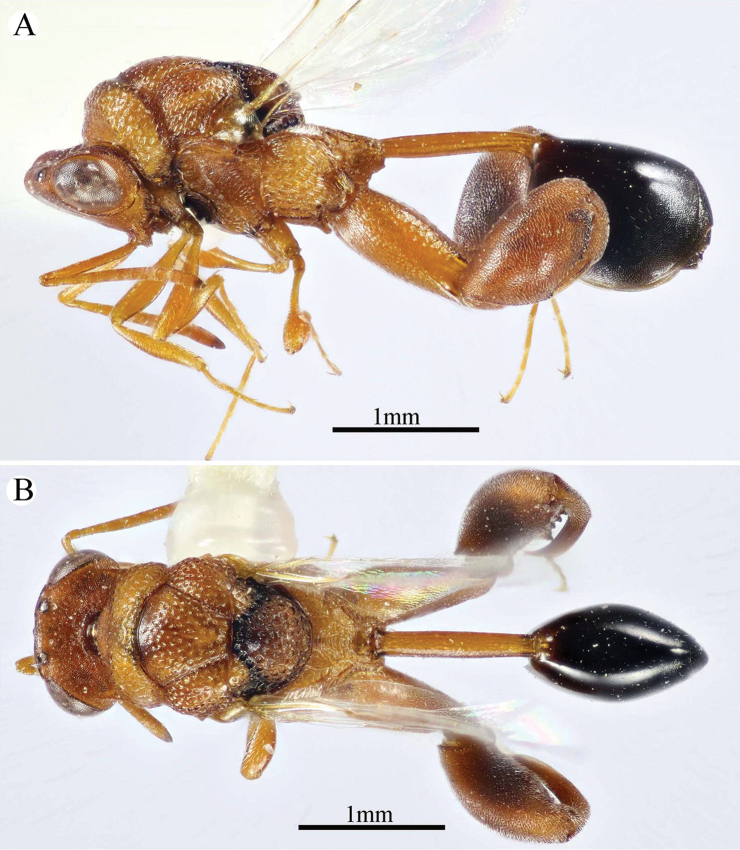
*Epitranus hamoni* complex (female, light form) **A, B** habitus (lateral and dorsal views respectively).

***Female*** (Figs 17–20). Body length ca. 3.15 mm; fore wing length ca. 2.0 mm. Body blackish brown, with the following parts are red to reddish brown (Figs 17A, B, 18A): head (except a black, broad lower band on occiput), pronotum, scapula, propodeum postero-laterally, metepimeron, gastral petiole, antenna and legs (dorsal face of metacoxa and outer face of metafemur with black tint). This species is diagnosed by the following combination of characters: Occiput densely reticulate, nearly bare (Fig. 18D); frontal lobe reduced to a faint transverse carina, thus exposing clypeus (Fig. 18B); interantennal projection represented by a low, but sharp lamina; flagellum somewhat clavate (Fig. 18C), ca. 0.93× as long as head width; anellus transverse, ca. 0.3× as long as wide; F1 as long as its width, ca. 0.9× as long as F2; F3 as long as wide; clava ca. 2.45× as long as wide. Interspaces between foveolae as well as their bottoms on mesosomal dorsum and pleura are densely reticulate (Fig. 18D); propodeum fairly dull, with areolae vague and finely punctate on their bottoms; median areola opened posteriorly, with lateral ridges short (not extending to meet transverse carina of adpetiolar areola) (Fig. 18D). Fore wing (Fig. 19A) with distinctly reduced pilosity, with scattered setae and microtrichiae on apical half of underside; STV reduced, gently sloping, forming with the anterior margin an angle of ca. 35°. Metafemur with a broad triangular tooth basoventrally followed by eight spaced teeth (Fig. 19B); tarsal scrobe on metatibia 0.6× as long as metatibial length, polished and reaching sub-basal extremely low hump that represents the sub-basal prominence (Fig. 19C). Metasomal petiole relatively long (5.7× as long as wide, 1.1× as long as dorsal length of Gt_1_, and 0.8× as long as gaster), dorsally with two longitudinal (sublateral and lateral) ridges, of which sublateral one ends slightly before apex of petiole, area between them flat and finely punctate (Fig. 19D); Gaster relatively short, 1.43× as long as high in profile (Fig. 17A).

**Figure 21.**
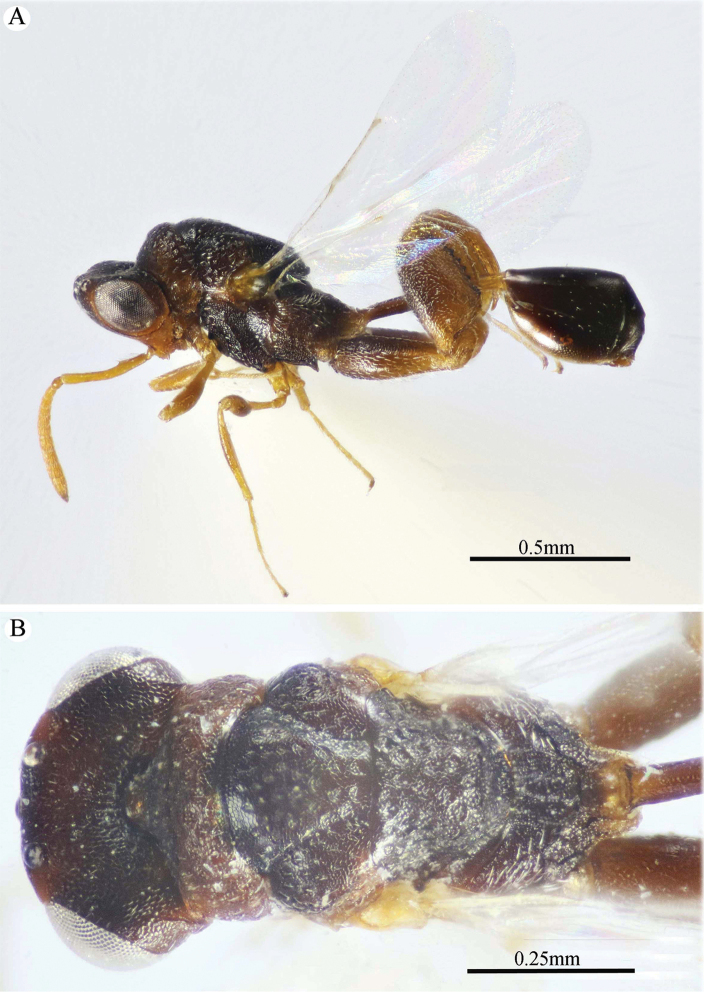
*Epitranus hamoni* complex (male, dark form) **A** habitus (lateral view) **B** head and mesosoma (dorsal view).

**Figure 22.**
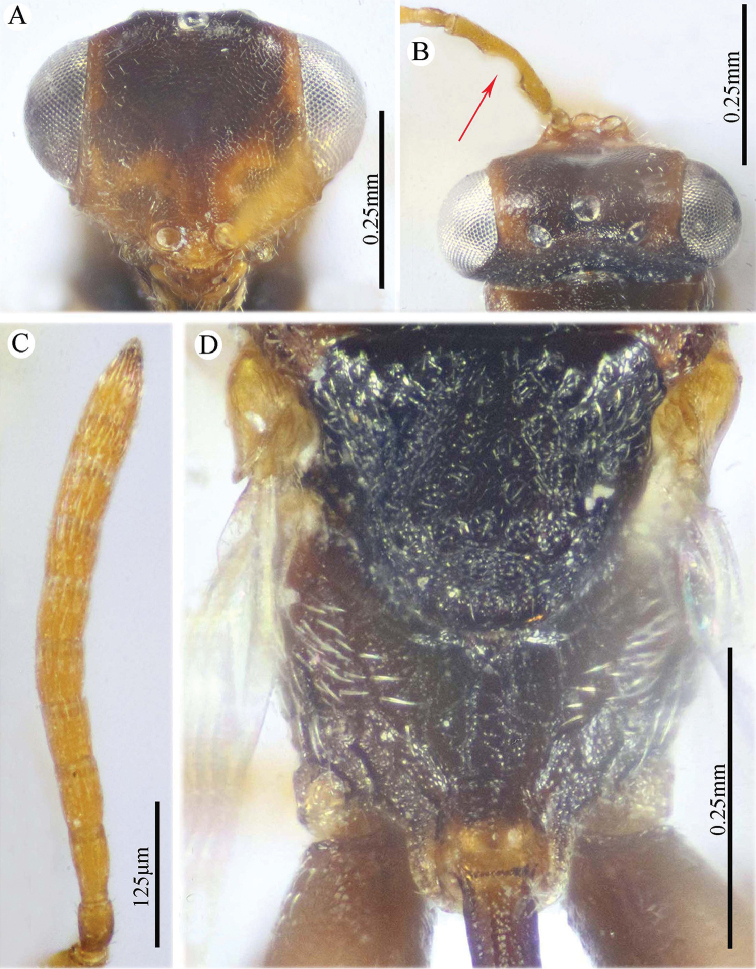
*Epitranus hamoni* complex (male, dark form) **A** head (frontal view) **B** head and antennal scape (dorsal view) **C** antennal pedicel and flagellum **D** mesoscutellum, metanotum & propodeum (dorsal view).

**Figure 23.**
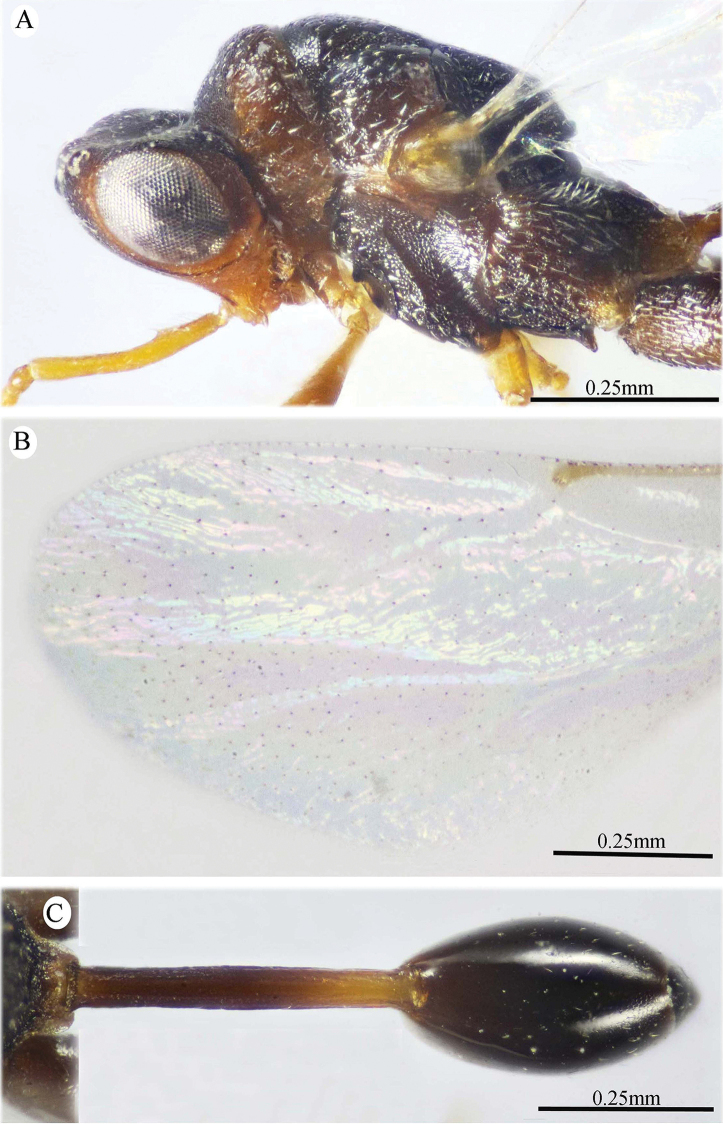
*Epitranus hamoni* complex (male, dark form) **A** head and mesonotum (lateral view) **B** apical part of fore wing **C** metasoma (dorsal view).

**Figure 24.**
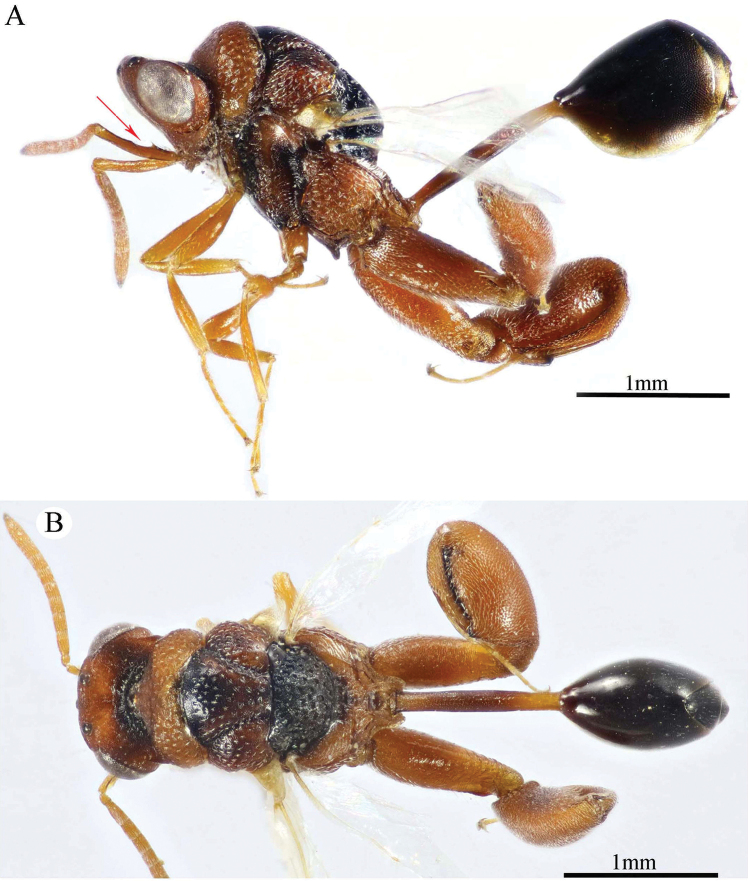
*Epitranus hamoni* complex (male, light form) **A, B** habitus (lateral and dorsal views respectively).

***Male*** (Figs 21–24). Similar to female except for: head and mesosoma generally dark brown to black, with inner margins of eye, lower half of face and pronotal lateral panel red, rest of the pronotum reddish brown (Figs 21A, B, 22A); interantennal projection absent (Fig. 22A); OOD short, ca. 1.43× as long as OD (Fig. 22B); scape of antenna with deep excavation nearly along its dorsal mesal third (Fig. 22B); flagellomeres slenderer than in female (Fig. 22C); foveolae on mesosomal dorsum sparser; propodeal median areola narrow, 4.0× as long as wide, reaching transverse carina of adpetiolar areola (Fig. 22D); petiole longer, ca. 8.0× as long as wide (Fig. 23C, 24B).

###### Remarks.

This species shows variation in color, some body sculpturing, and measurements among females and males as well. One of the three examined females, the body (including antennae and legs) is generally bright red, only darkened along the anterior and lateral sides of mesoscutellum, inner surface of metafemur, and gaster (Fig. 20A, B); in the other female specimens, body blackish brown, with the following parts are red to reddish brown (Figs 17A, B, 18A): head (except a black, broad lower band on occiput), pronotum, scapula, propodeum postero-laterally, metepimeron, gastral petiole, antenna and legs (dorsal face of metacoxa and outer face of metafemur with black tint). In the reddish specimen, the middle lobe of mesoscutum with denser and smaller setiferous punctures (Fig. 20B), mesoscutellum foveolate, with spaces less than a foveola diameter (ca. 0.5× diameter apart), bottom of foveolae smooth (Fig. 20B) (in the dark specimens, punctures on mesoscutum sparser and a little larger (Fig. 18D); mesoscutellum densely and deeply foveolate, without considerable interspaces between foveolae (Fig. 17B)); in the red specimen, petiole 6.3× as long as wide, 0.9× as long as gaster in dorsal view (Fig. 20B) (in the dark specimens, petiole ca. 5.7× as long as wide, 0.77× as long as gaster in dorsal view (Fig. 17B)); in the reddish specimen, posterior margin of Gt_1_ straight (Fig. 20B) (in the dark ones, posterior margin of Gt_1_ deeply concave (Fig. 17B)); in the reddish specimen, F1 ca. 2. 15× as long as wide, and distinctly longer than F7 (1.2×) (ca. 1.27× as long as wide, and slight shorter to as long as F7 in the dark specimens); STV obviously separated from anterior margin of the wing, making an angle of 45° in the light specimen (while in the darker specimens STV adheres to anterior margin of the wing making an angle of ca. 35°).

In the two examined males, one with the red color predominates, being seen in the head (except dark occiput) including antennae, pronotal collar, propodeum, legs (hind legs darker), and petiole (Fig. 24A, B); the other male specimen is nearly entirely dark brown to black, with inner margins of eye, lower half of face and pronotal lateral panel red (Fig. 21A, B). In the reddish specimen, mesoscutum sparsely setiferous punctate anteriorly, and sparsely foveolate posteriorly (Fig. 24B) (in the dark specimen superficially, sparsely foveolate throughout (Fig. 21B); in the reddish specimen, head asetose postero-laterally (Fig. 24B) (in the dark one, head densely setose postero-laterally (Fig. 21B)); in the reddish specimen metacoxa 2.6× as long as wide (Fig. 24B) (in the dark specimen, metacoxa 2.77× as long as wide (Fig. 21A); in the reddish specimen, petiole 9.3× as long as wide, and approximately as long as gaster middle length in dorsal view (Fig. 24B), 1.6× as long as gaster height in lateral view (Fig. 24A) (in the dark specimen, petiole 8.0× as long as wide, 1.12× as long as gaster in dorsal view (Fig. 23C), 2.4× as long as gaster height in lateral view (Fig. 21A)); in the reddish specimen, posterior margin of Gt_1_ deeply concave (Fig. 24B) (in the dark specimen, posterior margin of Gt_1_ straight (Fig. 23C)).

###### Hosts.

Unknown.

###### Material examined.

1♂, Kingdom of Saudi Arabia, Al-Baha, Al Mikhwa, Shada Al-Ala Natural Reserve [19°50'34.48"N, 41°18'39.44"E, Alt. 1681 m], sweeping net, 27.VII.2015, leg. Ahmed M. Soliman [KSMA]; 1♀, Kingdom of Saudi Arabia, Asir, Abha, Garf Raydah Natural Reserve [18°11'36.93"N, 42°23'25.17"E, Alt. 1772 m], sweeping net, 16.IV.2016, leg. Ahmed M. Soliman [KSMA]; 1♂, Kingdom of Saudi Arabia, Al-Baha, Al Mikhwa, Shada Al-Ala Natural Reserve [19°50'34.95"N, 41°18'40.04"E, Alt. 1679 m], sweeping net, 7.IV.2019, leg. Ahmed M. Soliman [KSMA]; 1♀, Kingdom of Saudi Arabia, Al-Baha, Al Mikhwa, Shada Al-Ala Natural Reserve [19°50'34.89"N, 41°18'39.43"E, Alt. 1689 m], sweeping net, 9.IV.2019, leg. Ahmed M. Soliman [KSMA]; 1♀, Kingdom of Saudi Arabia, Riyadh, Dirab Station of Research, [24°25'22.91"N, 46°39'15.02"E, Alt. 1689 m], Malaise trap, 19.VII−9.VIII.2020, leg. Ahmed M. Soliman [KSMA].

###### Distribution.

Burkina Faso (Risbec 1957; Noyes 2019), UAE (Delvare 2017), Saudi Arabia (Al-Baha and Riyadh regions) (new record).

##### Epitranus inops

Taxon classificationAnimaliaHymenopteraChalcididae

Steffan, 1957

3743AC83-84B8-590C-A881-D8FF98569C30

**Figure 25.**
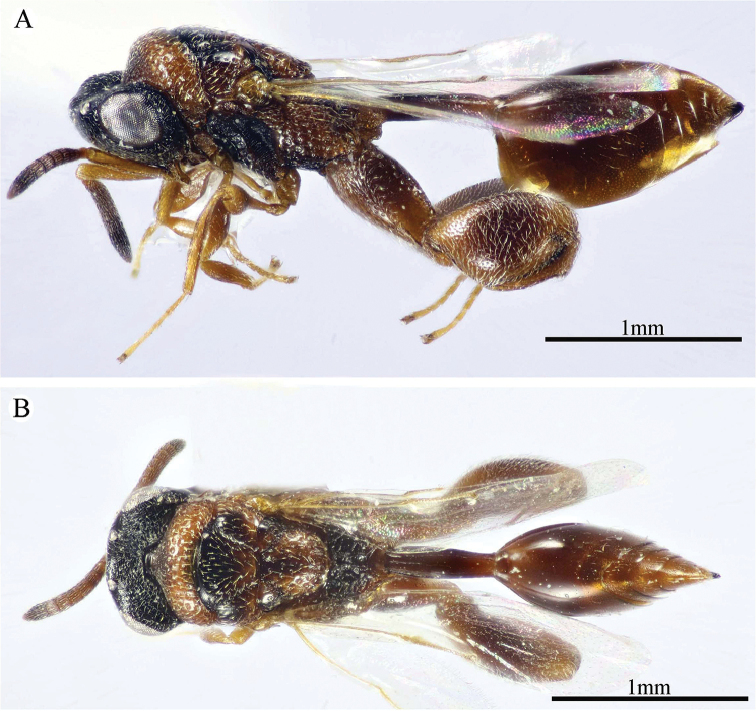
*Epitranus inops* Steffan (female) **A, B** habitus (lateral and dorsal views respectively).

**Figure 26.**
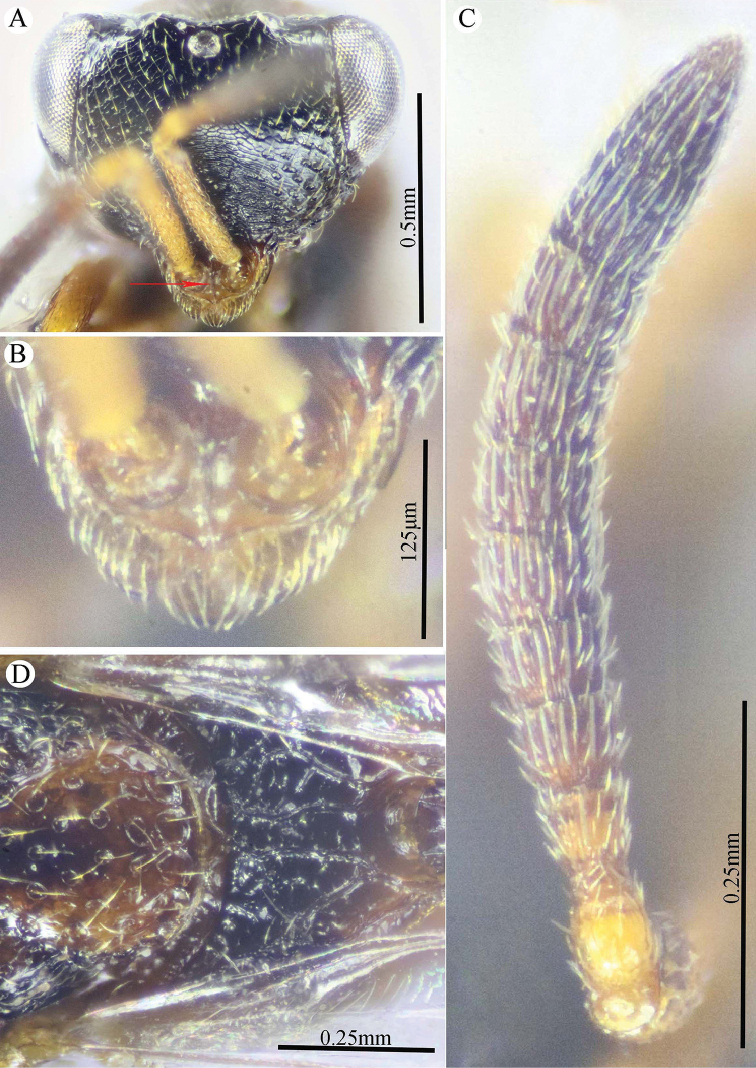
*Epitranus inops* Steffan (female) **A** head (frontal view) **B** lower part of face showing frontal lobe (frontal view) **C** antennal pedicel and flagellum **D** mesoscutellum and propodeum (dorsal view).

**Figure 27.**
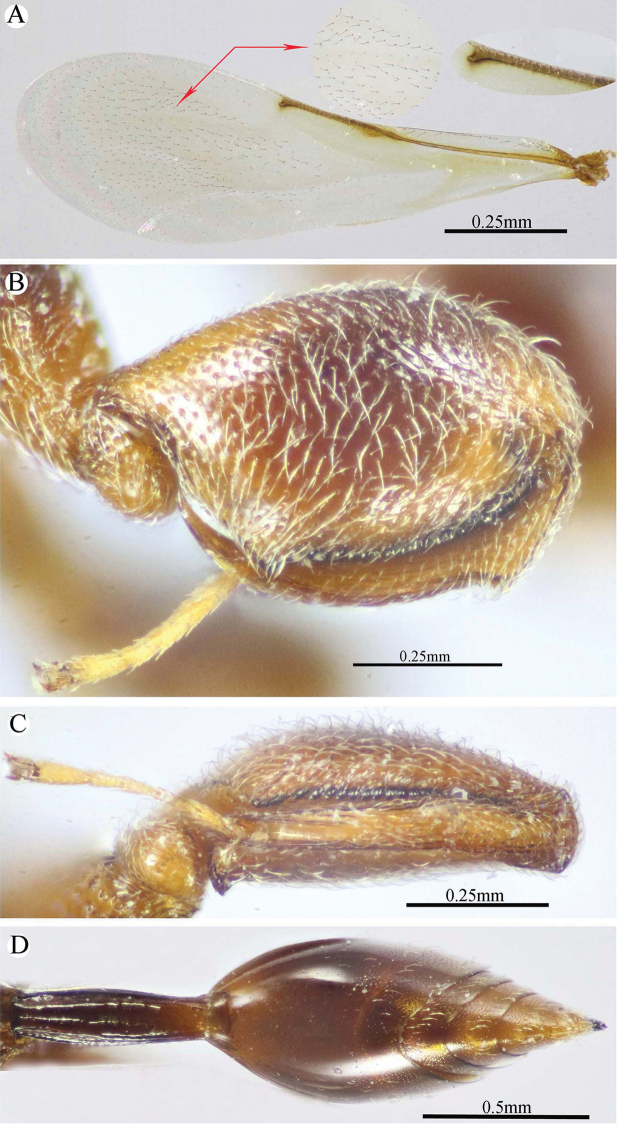
*Epitranus inops* Steffan (female) **A** fore wing (parts of wing membrane and MV and STV magnified) **B, C** hind leg, excluding coxa (outer and ventral views respectively) **D** metasoma (dorsal view).

Figures 25 , 26 , 27

Epitranus inops Steffan, 1957: 75, 86–88. Original description. ♀, ♂. Democratic Republic of Congo.
Epitranus inops Steffan,1957: Sauphanor et al. 1987: Ivory Coast: host.

###### Re-description.

***Female*** (Figs 25–27). Body length 3.4–3.9 mm. Fore wing length 2.1–2.5 mm. Head, except frontal lobe and antennal toruli, black (Fig. 26A); mesosoma reddish brown, with various extent of brownish, more or less dark, areas on mesoscutum, axilla, propodeum, mesopleuron, metepisternum anteriorly (Fig. 25B); tegula testaceous (Fig. 25A, B). This species is recognized by the following combination of characters: interantennal lamina present (Fig. 26A); frontal lobe moderately long (Fig. 26B); subantennal distance ca. 2.5× as long as interantennal distance, with two submedian indentations on ventral edge (Fig. 26B); supra antennal surface delimited laterally by faint step-like ridge; discal area faintly alutaceous, the network following curved lines, separated from inner orbit and median ocellus by four or five rows of moderately large punctures, interspaces between punctures smooth (Fig. 26A); preorbital groove vestigial dorsally, progressively thickened towards the suborbital groove; outline of frons slightly and regularly convex in dorsal view; funiculars, from F2, somewhat transverse (Fig. 26C); clava bi-segmented (Fig. 26C). Mesosoma hardly convex, with flattened mesoscutellum (Fig. 25A, B); setae on mesonotum thin, adpressed and longer than puncture diameter (Fig. 25B); propodeum dull, with numerous irregular rugae, median areola complete, with subparallel sides (Fig. 26D); adpetiolar areola with curved anterior carina (Fig. 26D). Fore wing (Fig. 27A) rather densely setose on apical half on underside; STV forming with anterior margin an angle of ca. 45°; metacoxal 2× as long as wide, with flattened outer dorsal side; metafemur serrulate behind the basal tooth (Fig. 27B); metatibial process only delimited posteriorly on inner side along tarsal scrobe, visible anteriorly through the presence of a wrinkle, tarsal scrobe far from reaching sub-basal prominence, the latter with four denticles on edge, visible solely from behind for being concealed by the pubescence (Fig. 27C); metasomal petiole 3.4–3.7× as long as wide, as long as or slightly shorter than dorsal length of Gt_1_ (0.95×), and 0.50–0.65× as long as gaster length, its sides hardly convex, with a weak median carina evanescent on apical third, sublateral and lateral ridges complete, the area between sublateral ridges smooth and shiny (Fig. 27D); gaster 1.55–1.70× as long as high (Fig. 25A).

***Male***. Similar to female except flagellum 1.2× head width; anellus ca. 0.3× as long as wide; F1 twice as long as wide; F7 subquadrate; gastral petiole slightly longer, 4.5× as long as wide (Steffan 1957).

###### Hosts.

The species was reared from stored yam together with *Euzopherodes vapidella* Man (Pyralidae), and other small moths (Sauphanor et al. 1987).

###### Material examined.

1♀: Kingdom of Saudi Arabia, Al-Baha, Al Mikhwa, Shada Al-Ala Natural Reserve [19°50'34.87"N, 41°18'40.04"E, 1686 m], sweeping net, 5.V.2015, leg. Ahmed M. Soliman [KSMA]; 1♀, Kingdom of Saudi Arabia, Al-Baha, Al Mikhwa, Shada Al-Ala Natural Reserve [19°50'34.89"N, 41°18'39.43"E, 1689 m], sweeping net, 9.IV.2019, leg. Ahmed M. Soliman [KSMA]; 1♀, Kingdom of Saudi Arabia, Asir, Abha, Garf Raydah Natural Reserve [18°11'40.98"N, 42°23'45.66"E, 1861 m], sweeping net, 12.IV.2019, leg. Ahmed M. Soliman [KSMA].

###### Distribution.

Democratic Republic of Congo (Zaire) (Steffan 1957), Ivory Coast (Sauphanor et al. 1987), Saudi Arabia (Al-Baha and Asir regions) (new record).

##### Epitranus torymoides

Taxon classificationAnimaliaHymenopteraChalcididae

(Risbec, 1953)

0973CE8B-3528-561B-9EEE-A0D24F0BCE8E

**Figure 28.**
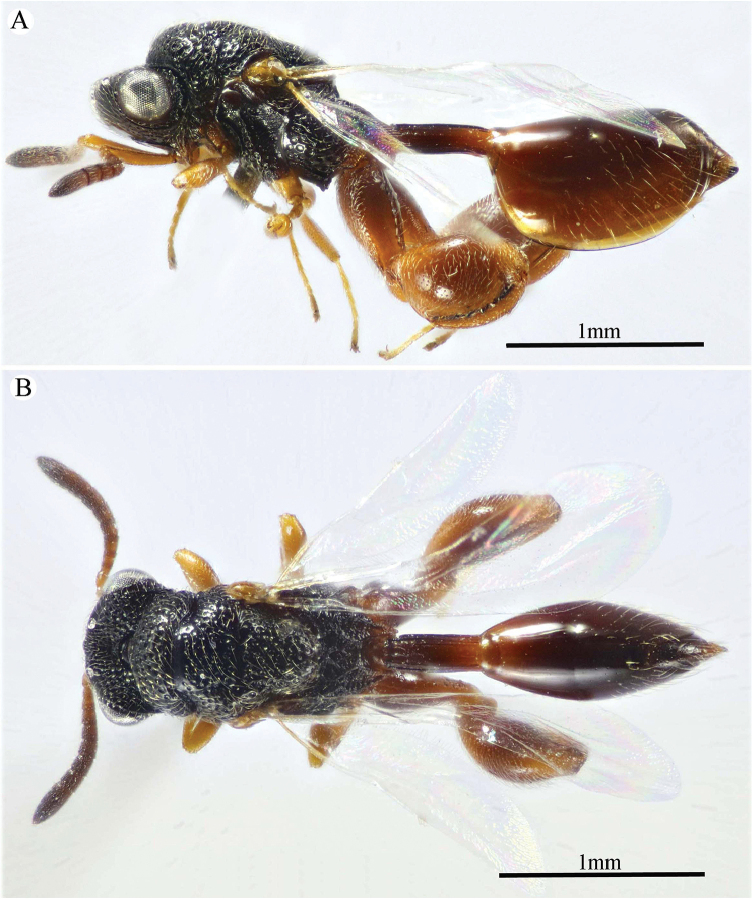
*Epitranus torymoides* (Risbec) (female) **A, B** habitus (lateral and dorsal views respectively).

**Figure 29.**
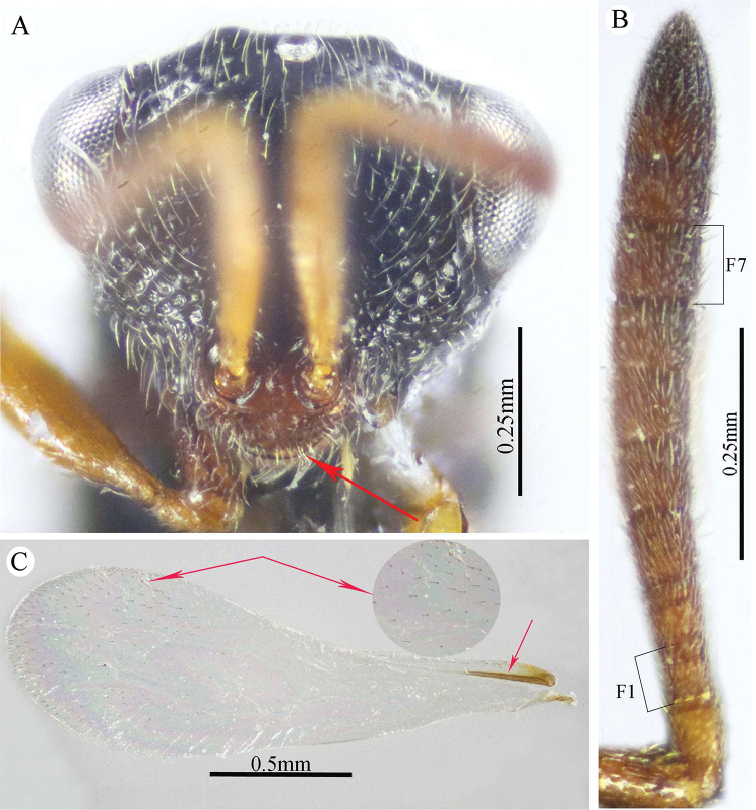
*Epitranus torymoides* (Risbec) (female) **A** head (frontal view) **B** antennal pedicel and flagellum **C** fore wing (part of wing membrane magnified).

**Figure 30.**
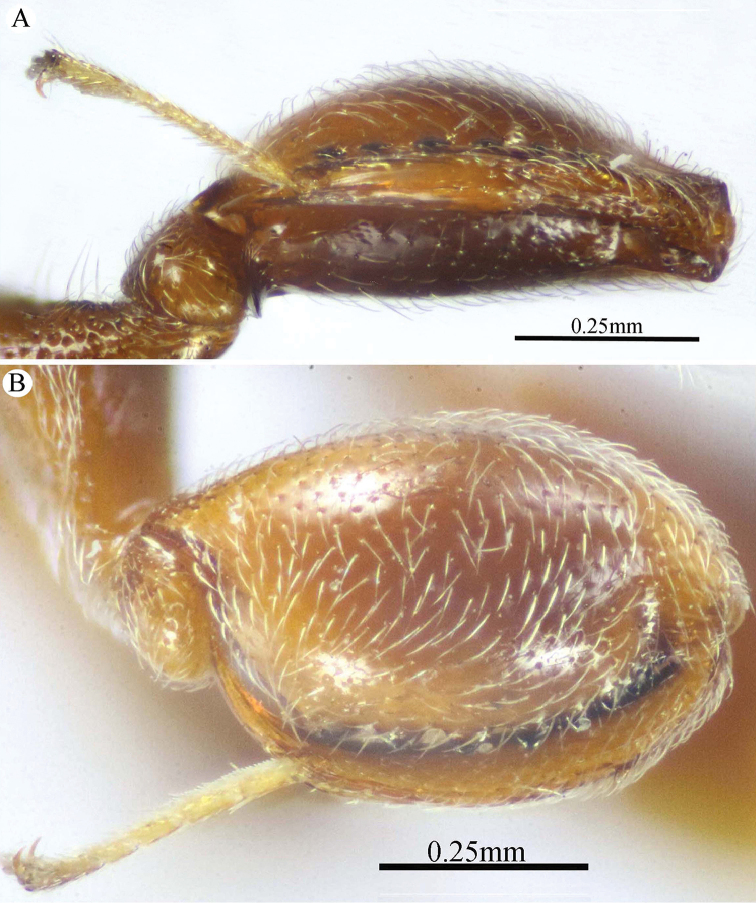
*Epitranus torymoides* (Risbec) (female) **A, B** hind leg, excluding coxa (outer and ventral views respectively).

Figures 28 , 29 , 30

Chalcitella torymoïdes Risbec, 1953: 591. Original description ♂. Ivory Coast.
Epitranus torymoides Risbec, 1953: Delvare, 2017: 244.

###### Re-description.

***Female*** (Figs 28–30). Body length 2.65–3.20 mm. Fore wing length 1.85–2.00 mm. Head, except reddish frontal lobe, and mesosoma black (Figs 28A, B, 29A); metasoma dark brown with slight reddish tint laterally on tergites and on sternites (Fig. 28A, B); fore and mid legs, metatrochanter, metatarsus, scape and tegula testaceous (Fig. 28A); pedicel, flagellum and metafemur dark brown (Fig. 28A, B). This species is recognized by the following combination of characters: frons laterally and dorsally, and dorsum of mesosoma, with moderately long, suberect and thin setae (Figs 28B, 29A); interantennal projection absent (Fig. 29A); frontal lobe moderately protruding, with subantennal distance ca. 1.3× as long as interantennal distance, ventral edge of projection broadly rounded, entire (Fig. 29A); supra antennal surface smooth, completely delimited by step-like margin, 1.7× as high as wide; discal area reduced to a smooth crescentic surface above the supra antennal one; rest of the frons with moderately large setiferous punctures (Fig. 29A); gena areolate (Fig. 28A); preorbital, suborbital and postorbital grooves well impressed, the first one smooth, while the others areolate; postorbital carina joining genal carina at a level slightly below ventral edge of eye; outline of frons hardly and regularly convex in dorsal view; flagellum strongly clavate, 0.96× as long as head width (Fig. 29B); the two basal flagellomeres subquadrate, lacking MPS; clava bi-segmented (Fig. 29B); pronotum and mesonotum densely and regularly punctured (Fig. 28B); pronotal collar rounded on dorsum (Fig. 28B); propodeal surface with numerous secondary rugae, with fusiform median areola not quite reaching the truncate adpetiolar areola (Fig. 28B). Fore wing (Fig. 29C) with strongly reduced venation, only base of SMV present; apical half of wing membrane with scattered setae on underside; metacoxa with nearly smooth and flattened outer dorsal side; metafemur with seven or eight widely spaced teeth following the stout basal one on ventral margin (Fig. 30B); metatibial process only delimited on inner side along the short tarsal groove, the latter approximately one third, the rest of the process is visible as being sparsely and finely setose, the setation not concealing the integument surface there (Fig. 30A); sub-basal prominence vestigial, hardly visible, with a single denticle (Fig. 30A); metasomal petiole 2.8–3.0× as long as wide, 0.6× as long as dorsal length of Gt_1_, and 0.40–0.45× as long as gaster (Fig. 28B); the area between sublateral ridges on petiole flat and rough, with hardly indicated longitudinal median carina at base and apex, absent medially (Fig. 28A, B); Gaster 1.8× as long as high (Fig. 28A).

***Male***. Differs from female in the following: flagellum and metasomal petiole darker, dark brown to black, the latter reddish brown posteriorly; metasoma with black tint dorsally; anellus transverse; flagellum slender, F1 ca. 2× as long as wide, 1.28–1.30× as long as each of F2 and F7; petiole with a complete median carina.

###### Hosts.

Unknown.

###### Material examined.

1♀: Kingdom of Saudi Arabia, Al-Baha, Al Mikhwa, Shada Al-Ala Natural Reserve [19°50'34.87"N, 41°18'40.04"E, 1686 m], sweeping net, 5.V.2015, leg. Ahmed M. Soliman [EFC]; 1♂: Kingdom of Saudi Arabia, Asir, Muhayil, Wadi Sabean [18°17'53"N, 42°07'39"E, 775 m], Sucking device, 10.II.2016, leg. A. Al-Ansi [KSMA]; 7♀&1♂: Kingdom of Saudi Arabia, Asir, Muhayil, Wadi Heli [18°30'10.66"N, 42°01'56.07"E, 450 m], sweeping net, 23.X.2016, leg. Ahmed M. Soliman [KSMA]; 1♂: Kingdom of Saudi Arabia, Jazan, Damad, Al Shuqayri [17°07'39.50"N, 42°48'44.88"E, 90 m], Sucking device, 12.V.2018, leg. Ahmed M. Soliman [KSMA]; 1♀: Saudi Arabia, Asir, Abha, Garf Raydah Natural Reserve [18°11'40.98"N, 42°23'45.66"E, 1861 m], sweeping net, 12.IV.2019, leg. Ahmed M. Soliman [KSMA].

###### Distribution.

Côte d’Ivoire (Risbec 1953), Saudi Arabia (Al-Baha, Asir, and Jazan regions) (new record).

**Figure 31.**
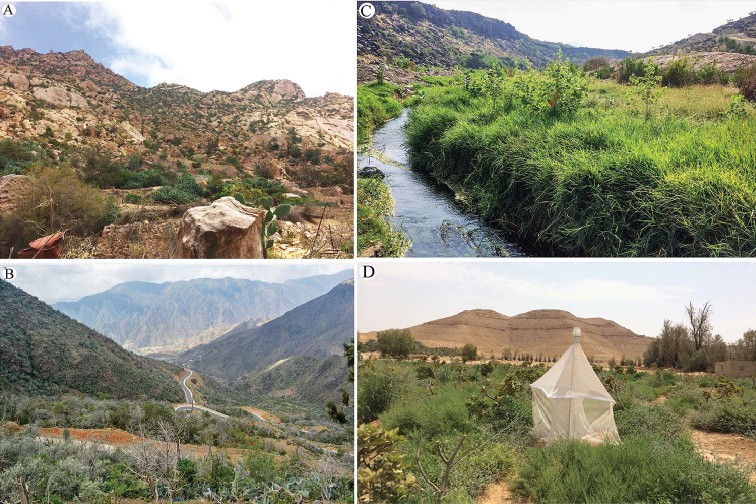
Examples of the habitat where the *Epitranus* species were collected **A** Shada Al-Ala Natural Reserve (Al-Baha) **B** Garf Raydah Natural Reserve (Asir) **C** Wadi Heli (Asir) **D** Dirab Station of Research (Riyadh).

## Discussion

Epitraninae are native from the Old World were probably accidentally introduced in the New World before cautionary measures were made. Their presence in some parts of the New World countries as Caribbean islands and Brazil, pre-1900, strongly suggests their introduction via maritime ports.

The Afrotropical species of the genus *Epitranus* Walker were revised by Schmitz (1946, under *Anacryptus*) and Steffan (1957), who provided keys and described many new species. In addition, some sporadic studies who included descriptions of *Epitranus* species among other chalcidids (Westwood 1835; Walker 1862; Ruschka 1924; Masi 1940, 1943; Risbec 1953, 1957). Approximately 38% of the total number of species of the world possess Afrotropical affinities (see Noyes 2019). Since Steffan (1957), no further revisions covered the Afrotropical *Epitranus*.

In the Arabian Peninsula, *Epitranus* was recorded by one study (Delvare 2017), which reported two species, *E. hamoni* and *E. torymoides*, both in the United Arab Emirates.

The present study supplies new information in the Arabian Peninsula, and the first for Saudi Arabia. Here we reported four new records, *E. clavatus*, *E. hamoni* complex, *E. inops*, and *E. torymoides*, all of them with Afrotropical distribution. Three new species are also described and illustrated, *E. delvarei*, *E. similis*, and *E. subinops*, thus raising the total number in the whole Arabian Peninsula to seven species.

Little is known about the biology of the Afrotropical species of the genus *Epitranus*, from what is known from a single species, *E. inops*. It was reared from stored yam together with the pyralid moth, *Euzopherodes vapidella*, and other small moths (Sauphanor et al. 1987).

All species under study were collected from Al-Baha, Asir, Jazan, and Riyadh provinces (southwestern and central regions of Saudi Arabia). Consequently, the area under study (southwestern Saudi Arabia) should be included in the Afrotropical realm (see Gadallah and Brothers 2020), and this is closely correlated with the floristic composition of this area, thus supporting many of other previous works (El-Hawagry et al. 2013, 2015; Sharaf et al. 2014; Gadallah et al. 2018; Gadallah and Brothers 2020).

However, more species are expected to occur because of the biodiversity richness of the country, as it occupies the major part of the Arabian Peninsula (Aldhebiani and Howladar 2015). For this reason, more collection trips and studies are necessary to clarify the distributions as well as the host records of this interesting genus in other parts of Saudi Arabia.

## Supplementary Material

XML Treatment for Epitranus delvarei

XML Treatment for Epitranus similis

XML Treatment for Epitranus subinops

XML Treatment for Epitranus clavatus

XML Treatment for Epitranus hamoni

XML Treatment for Epitranus inops

XML Treatment for Epitranus torymoides

## References

[B1] Aldhebiani AY Howladar SM (2015) Floristic diversity and environmental relations in two valleys, south west Saudi Arabia. International Journal of Science and Research 4(2): 1916–1925. https://www.ijsr.net/get_abstract.php?paper_id=SUB151587

[B2] Ashmead WH (1904) Classification of the chalcid flies of the superfamily Chalcidoidea, with descriptions of new species in the Carnegie Museum, collected in South America by Herbert H. Smith. Memoirs of the Carnegie Museum 1(4): 225–551. https://www.biodiversitylibrary.org/page/10924545

[B3] Bouček Z (1982) Oriental chalcid wasps of the genus *Epitranus*. Journal of Natural History 16: 577–822. 10.1080/00222938200770451

[B4] Bouček Z (1988) Australasian Chalcidoidea (Hymenoptera). A Biosystematic Revision of Genera of Fourteen Families, with a Reclassification of Species. CAB International Wallingbord, Oxon, Cambrian News Ltd; Aberystwyth, Wales, 832 pp https://www.cabdirect.org/cabdirect/abstract/19881109893

[B5] Burks BD (1936) The Nearctic Dirhinini and Epitranini (Hymenoptera: Chalcididae). Proceedings of the National Academy of Sciences of the United States of America, Washington 22: 283–287. 10.1073/pnas.22.5.283 PMC1076761 16577712

[B6] Cameron P (1883) Descriptions of new genera and species of Hymenoptera. Transactions of the Entomological Society of London 1883: 187–197. 10.1111/j.1365-2311.1883.tb02945.x

[B7] Cruaud A Delvare G Nidelet S Sauné L Ratnasingham S Chartois M Blaimer BB Gates M Brady SG Faure S Noort van S Rossi J-P Rasplus J-Y (2020) Ultra-Conserved Elements and morphology reciprocally illuminate conflicting phylogenetic hypotheses in Chalcididae (Hymenoptera, Chalcidoidea). Cladistics 0: 1–35. 10.1101/761874 34478176

[B8] Delvare G (2017) Order Hymenoptera, family Chalcididae. Arthropod fauna of the UAE 6: 225–274 https://agritrop.cirad.fr/586551/1/Delvare%202017%20Fauna%20UAE%20%20Chalcididae.pdf

[B9] El-Hawagry MS Khalil MW Sharaf MR Fadl HH Aldawood AS (2013) A preliminary study on the insect fauna of Al-Baha Province, Saudi Arabia, with descriptions of two new species. ZooKeys 274: 1–88. 10.3897/zookeys.274.4529 PMC3677392 23794807

[B10] El-Hawagry MS Sharaf MR Al-Dhafer MH Fadl HH Aldawood AS (2015) Addenda to the insect fauna of Al-Baha Province, Kingdom of Saudi Arabia with zoogeographical notes. Journal of Natural History 50(19–20): 1209–1236. 10.1080/00222933.2015.1103913

[B11] Fabricius JC (1804) Systema Piezatorum 2: 1–440. A.C. Reichard, Brunsvigae [Richards: Transactions of the Royal Entomological Society of London 83: 144. https://www.biodiversitylibrary.org/page/11001084 [publ. 1804 (1805 latest): Hedicke, Mitteilungen der Entomologischen Gesellschaft 10: 82–83, text publ. 1804]

[B12] Gadallah NS Brothers DJ (2020) Biodiversity of aculeate wasps (Hymenoptera: Aculeata) of the Arabian Peninsula: Overview. Zootaxa 4754(1): 8–16. 10.11646/zootaxa.4754.1.4 32230209

[B13] Gadallah NS Soliman AM Abu El-Ghiet UM Elsheikh TY Al Dhafer HM (2018) The family Leucospidae (Hymenoptera: Chalcidoidea) from the South of Saudi Arabia, with the first report of the genus *Micrapion* and description of *Leucospis arabica* sp. nov. Journal of Natural History 52(31–32): 2071–2096. 10.1080/00222933.2018.1510557

[B14] Girault AA (1913) Some chalcidoid Hymenoptera from North Queensland. Archiv für Naturgechichte (A) 79(6): 70–90. 10.4039/Ent4742-2

[B15] Girault AA (1914) A new chalcid genus and species of Hymenoptera from Australia. Entomological News 25: 30. https://www.biodiversitylibrary.org/page/2628628

[B16] Girault AA (1915) Australian HymenopteraChalcidoidea-XIV. The family Chalcididae with description of new genera and new species. Memoirs of the Queensland Museum 4: 314–365. https://www.biodiversitylibrary.org/page/13217352

[B17] Habu A (1960) A revision of the Chalcididae (Hymenoptera) of Japan with description of sixteen new species. Bulletin of National Institute of Agricultural Sciences, Tokyo (c) 11: 131–363.

[B18] Harris RA (1979) A glossary of the surface sculpturing. Occasional Papers of Entomology, California Department of Food and Agriculture 28: 1–31. https://zenodo.org/record/26215#.X4DTUdAzbIU

[B19] Heraty JM Burks RA Cruaud A Gibson GA Liljeblad J Munro J Rasplus JY Delvare G Janšta P Gumovsky A Huber J et al. (2013) A phylogenetic analysis of the megadiverse Chalcidoidea (Hymenoptera). Cladistics 29(5): 466–542. 10.1111/cla.12006 34798768

[B20] Husain T Agarwal MM (1982) Taxonomic studies on Indian Epitraninae (Hymenoptera: Chalcididae). Oriental Insects 15(4): 413–432. 10.1080/00305316.1981.10434340

[B21] Mani MS Dubey OP Kaul BK Saraswat GG (1973) On some Chalcidoidea from India. Memoirs of the School of Entomology, St. John’s College, Agra, No. 2: 30–31.

[B22] Masi L (1917) Chalcididae of Sychelles islands. (With an appendix by J.J. Kieffer), Novitates Zoologicae 24: 121–230. 10.5962/bhl.part.23148

[B23] Masi L (1933) H. Sauters Formosa-Ausbeute. Chalcididae (Hym.). II Teil. Konowia 12: 1–18. https://www.nhm.ac.uk/resources/research-curation/projects/chalcidoids/pdf_X/Masi933b.pdf

[B24] Masi L (1940) Descrizioni di Calcididi raccolti in Somalia dal. Prof. G. Russo con note sulle species congeneri. Bollettino del R. Laboratoria di Entomologia Agraria di Pertici 3: 247–324. https://www.nhm.ac.uk/resources/research-curation/projects/chalcidoids/pdf_X/Masi940c.pdf

[B25] Masi L (1943) Nuove specie di imenotteri calcididi Diagnosti precentive. Missione biologica Sagan-Omo diretla dal Prof. E. Zavattari. Bollottino della Società Entomologica Italiana 75: 65–68. https://www.nhm.ac.uk/resources/research-curation/projects/chalcidoids/pdf_X/Masi943.pdf

[B26] Moravvej SA Lotfalizadeh H Shishehbor P (2018) On the presence of the subfamily Epitraninae (Hymenoptera: Chalcidoidea, Chalcididae) in Iran. North-Western Journal of Zoology 14(2): 267–268

[B27] Narendran TC (1989) Oriental Chalcididae (Hymenoptera: Chalcidoidea). Zoological Monograph. Department of Zoology, University of Calicut Kerala, India, 1–441. https://archive.org/details/OrientalChalcididae

[B28] Narendran TC van Achterberg C (2016) Revision of the family Chalcididae (Hymenoptera, Chalcidoidea) from Vietnam, with the description of 13 new species. ZooKeys 576: 1–202. 10.3897/zookeys.576.8177 PMC4829922 27110185

[B29] Noyes J (2019) Universal Chalcidoidea Database. World Wide Web electronic publication. http://www.nhm.ac.uk/chalcidoids

[B30] Rasplus J-Y (1993) L’entomofaune de termitière morte de *Macrotermes*. Description d’une nouvelle espèce de *Spalangia* et note sur une espèce rare d’*Epitranus* (Hymenoptera, Pteromalidae, Chalcididae). Revue Française d’Entomologie (Nouvelle Série) 15(2): 81–84.

[B31] Risbec J (1953) Chalcidoides et proctotrupoides de l’Afrique occidentale Française (2^e^ supplement). Bulletin de l’Institut Français d’Afrique Noire 15: 549–809.

[B32] Risbec J (1957) Chalcidoides et proctotrupoides de l’Afrique occidentale Française (5^e^ supplement). Bulletin de l’Institut Français d’Afrique Noire (A) 19: 228–267.

[B33] Ruschka F (1924) Wissenschaftlische ergebnisse der Unterstützung de Akademie der Wissenschaften in Wien aus der Erbschaft treitl von F. Werner Unternommenen Zoologischen expedition nach dem Anglo-Ägyptischen Sudan (Kordofan) 1914. XIII. Hymenoptera (Chalcididae). Denkschriften der Akademie der Wissenschaften Wien. Mathematisch-Naturwissenschaften Klasse 99: 99–100. https://www.nhm.ac.uk/resources/research-curation/projects/chalcidoids/pdf_X/Ruschk924c.pdf

[B34] Sauphanor B Bordat D Delvare G Ratnadass A (1987) Insects of stored yams in the Ivory Coast. Faunistic inventory and biological data. L’Agronomie Tropicale, Nogent-sur-Mame 42(4): 305–312.

[B35] Schmitz G (1946) Chalcididae (HymenopteraChalcidoidea). Exploration du Parc National Albert. Mission G.F. de Witte (1933-1935) 48: 1–191. https://www.nhm.ac.uk/resources/research-curation/projects/chalcidoids/pdf_X/Schmit946.pdf

[B36] Sharaf MR Fischer BL Aldawood AS (2014) First record of the Myrmicine ant genus *Meranoplus* Smith, 1853 (Hymenoptera: Formicidae) from the Arabian Peninsula with description of a new species and notes on the zoogeography of the southwestern Kingdom of Saudi Arabia. PLOS One: e111298. 10.1371/journal.pone.0111298 PMC4222904 25375104

[B37] Steffan J-R (1957) Epitraninae (Hym. Chalcididae) du Musée Royal du Congo Belge. Revue de Zoologie et de Botanique Africaines 56(1/2): 71–91.

[B38] Walker F (1834) Monographia Chalciditum. (Continued). Entomological Magazine 2(1): 13–39. https://www.biodiversitylibrary.org/page/25333985

[B39] Walker F (1862) Notes on the chalcidites, and characters of undescribed species. Transactions of the Entomological Society of London 3(1): 345–397. https://www.biodiversitylibrary.org/page/32121299

[B40] Westwood JO (1835) Various hymenopterous insects from the collection of the Rev. F.W. Hope. Proceedings of the Zoological Society of London 3: 68–72. https://antcat.org/references/129806

